# Metabolomics and the Multi-Omics View of Cancer

**DOI:** 10.3390/metabo12020154

**Published:** 2022-02-07

**Authors:** David Wishart

**Affiliations:** 1 Department of Biological Sciences, University of Alberta, Edmonton, AB T6G 2E9, Canada; 2 Department of Computing Science, University of Alberta, Edmonton, AB T6G 2E8, Canada; 3 Department of Laboratory Medicine and Pathology, University of Alberta, Edmonton, AB T6G 2B7, Canada; 4 Faculty of Pharmacy and Pharmaceutical Sciences, University of Alberta, Edmonton, AB T6G 2H7, Canada; dwishart@ualbera.ca; Tel.: +1-780-492-8574

**Keywords:** cancer, metabolomics, exposome, genome, metabolome, metabotypes, oncometabolites

## Abstract

Cancer is widely regarded to be a genetic disease. Indeed, over the past five decades, the genomic perspective on cancer has come to almost completely dominate the field. However, this genome-only view is incomplete and tends to portray cancer as a disease that is highly heritable, driven by hundreds of complex genetic interactions and, consequently, difficult to prevent or treat. New evidence suggests that cancer is not as heritable or purely genetic as once thought and that it really is a multi-omics disease. As highlighted in this review, the genome, the exposome, and the metabolome all play roles in cancer’s development and manifestation. The data presented here show that >90% of cancers are initiated by environmental exposures (the exposome) which lead to cancer-inducing genetic changes. The resulting genetic changes are, then, propagated through the altered DNA of the proliferating cancer cells (the genome). Finally, the dividing cancer cells are nourished and sustained by genetically reprogrammed, cancer-specific metabolism (the metabolome). As shown in this review, all three “omes” play roles in initiating cancer. Likewise, all three “omes” interact closely, often providing feedback to each other to sustain or enhance tumor development. Thanks to metabolomics, these multi-omics feedback loops are now much more evident and their roles in explaining the hallmarks of cancer are much better understood. Importantly, this more holistic, multi-omics view portrays cancer as a disease that is much more preventable, easier to understand, and potentially, far more treatable.

## 1. Introduction

Cancer is a disease of many disguises. It can grow quickly or emerge slowly, it can be benign or malignant, it can strike at any age, and it can appear in almost any cell, tissue, or organ. To date, more than 200 different types of cancers have been named or identified [1] and the list keeps growing. The many faces of cancer have made it a difficult disease to describe and an even more difficult disease to understand. As a result, many theories have emerged to explain the origins of cancer and, even today, new theories continue to be advanced to explain how this “emperor of all maladies” [2] manifests itself. Interestingly, many of these theories on cancer and carcinogenesis have emerged in tandem with new advances in medical technology or new discoveries in biology or physiology.

Prior to the advent of modern medicine, cancer was viewed as a disease that arose from bad biofluid “humors”, or an unbalanced level of black bile in the body [3]. This was a concept that largely grew from the teachings of Hippocrates and persisted through the Middle Ages and early Renaissance. By the 1700s, as new understandings about the human circulatory system arose, the lymph theory of cancer emerged. The lymph theory posited that cancer was composed of fermenting lymph, varying in density, acidity, and alkalinity and that tumors grew from lymph constantly thrown out by the blood [3]. With the advent of epidemiology and new insights into contagions and how disease spreads, many physicians in the 18th century believed that cancer was either familial or contagious as it seemed to be passed among family members [3,4]. However, that idea soon faded and by the 1800s, with the widespread use of microscopes and the discovery of cells, the blastema theory of cancer emerged. This theory, which still persists today, suggested that cancer grew from certain progenitor cells (blastema) in the body [3,4]. With the introduction of anesthetics in the early 1800s, the rise of surgery in the mid 1800s, and a better understanding of physiology in the late 1800s, other theories emerged suggesting that cancer arose from chronic irritation or trauma to certain tissues or organs. By the early 1900s, as the effects of the industrial revolution were leading to higher rates of cancer, the first evidence that cancer could arise from chemical toxins (coal tar) was presented [3,4]. With the discovery of viruses in the early 20th century, it was shown shortly thereafter, that certain viruses (the Rous sarcoma virus) could also induce cancer [5]. As knowledge and interest in metabolism, nutrition, and biochemistry grew in the 1920s, Otto Warburg showed that cancer was primarily due to dysregulated metabolism leading to aerobic glycolysis [6]. With continuing advances in genetics along with the identification of cancers occurring in twins in the late 1940s [7], the view that cancer was a genetic disease began to take hold. As molecular biology gained ascendency in the 1970s, the discovery of oncogenes and tumor suppressor genes, in 1970, led to the development of a more refined somatic mutation theory (SMT) of carcinogenesis [8]. At about the same time, the discovery of stem cells in 1960s led to the emergence of the stem theory of cancer [9], which proposes that tumors contain a small number of stem cells that reproduce themselves to sustain the growth and spread of cancer. With the emergence of metabolomics in the early 2000s and the discovery of oncometabolites [10], the role of cellular metabolism and endogenous metabolites in carcinogenesis (i.e., the “black bile” and “fermenting lymph”) once again ascended into credibility. In some respects, it appears that our theories on cancer have nearly come full circle.

Almost all these ideas on the origins of cancer, including some of the oldest, have some merit. Additionally, almost all of these theories can be clustered into three conceptual groups. Some, such as the humor/bile, lymph, Warburg, and oncometabolite theories suggest that cancer is largely a metabolic disease or a disorder of the internal environment (i.e., arising from an altered metabolome). These metabolome-based theories highlight dysregulated metabolism and replicative immortality that are common hallmarks of all cancers [11]. Others, such as the trauma, irritation, contagion, or chemical toxin theories argue that cancer is really a disease primarily resulting from adverse effects of the external environment (i.e., arising from adverse effects of the exposome). These exposome-related theories emphasize the role of chemicals or microbes leading to tumor promoting inflammation, genomic instability, and sustained proliferative signaling in cancer [11]. Other theories, such as the blastema, familial, stem, and SMT theories of carcinogenesis suggest that cancer is a disease that is largely genetic in origin (i.e., arising from mutations in the genome). These genome-based theories highlight the role of genes and genetic instability leading to metastasis, immune avoidance, evading growth suppression, and sustained growth signaling in cancer [11].

The diversity of theories, their limited ability to explain all the hallmarks of cancer, and their disconnectedness have led to the emergence of various “camps” within the cancer research community. Unfortunately, none of these camps seems particularly interested in working with any of the other camps or in using their unique knowledge or insights to more fully understand cancer. As might be expected, the vast majority of cancer researchers fall into the genome camp, with more than 83,000 papers a year (PubMed query: (cancer) AND ((genetics) OR (genomics) OR (genome) OR (gene)) being published on cancer genetics or cancer genomics. A smaller number of cancer researchers fall into the exposome camp with about 19,000 papers a year being published on the effects of exposures (viral, bacterial, and mutagens) on cancer and carcinogenesis (PubMed query: (cancer) AND (pollutant) OR (mutagen) OR (carcinogen) OR (exposome) OR (viral) OR (bacterial)). The fewest number of cancer researchers fall into the metabolome camp, with just over 2000 papers a year being published on cancer metabolism or cancer metabolomics (PubMed query: (cancer) AND ((metabolomics) OR (metabolome)).

As evidenced from the number of published scientific papers, the genomic perspective on cancer has come to almost completely dominate the field. However, it is becoming increasingly clear that this genome-only view is incomplete [12,13]. In particular, it only explains the origins of a small fraction of known cancers, and it does not fully rationalize all of the hallmarks of cancer [11,13]. The genome-only perspective also tends to portray cancer as a disease that is inevitable (i.e., highly heritable), difficult to understand, and hard to treat [14]. New evidence shows that cancer is not only a genetic disorder, but also an environmental disorder, one that is often initiated by the external environment (the exposome), and then, sustained by the internal chemical environment (the metabolome and the epigenome). This multi-omics view, which integrates the effects of the genome, the exposome, and the metabolome together, appears to explain most of the hallmarks of cancer as well as cancer’s many disparate origins. More appealingly, it also portrays cancer as a disease that is much more preventable, easier to understand, and potentially far more treatable [13,14].

The purpose of this short review is to briefly discuss the three different perspectives of cancer: (1) cancer as a genetic disease (a disorder of the genome); (2) cancer as an environmental disease (a disorder of the exposome); and (3) cancer as a metabolic disease (a disorder of the metabolome) and to provide useful, quantitative data to support all three perspectives. In addition, new data are presented, showing that cancer is far less heritable or “genetic” than previously thought and how environmental, dietary, or metabolic effects contribute far more to cancer incidence and prevalence than previously realized. This review also shows how the exposome, the metabolome, and the genome interact closely, through several feedback loops, to not only initiate cancer but to also sustain cancer. The ultimate goal of this review is to encourage cancer researchers to adopt a more holistic, multi-omics view on cancer. It is hoped that this will lead to an improved appreciation of the disease as well as a better understanding of how to prevent and treat it.

## 2. Cancer as a Genetic Disease (the Genome View)

For more than three centuries it has been known that cancers run in families [3]. However, it was not until the late 1940s and early 1950s that a clear genetic or hereditary link for cancer was made through the analysis of twin registries and large family cohorts [7,15]. Familial cancer syndromes (or hereditary cancers) are germline cancers arising from a specific oncogenic mutation being passed from one generation to another [16]. More than 50 hereditary forms of cancer are known, including Lynch syndrome, familial adenomatous polyposis, hereditary breast and ovarian cancer, and Fanconi’s anemia. The existence of familial cancer syndromes or germline cancers provides the strongest evidence of the role of genetics and genetic mutations in cancer. Furthermore, many of the mutated genes identified in hereditary cancers or familial cancer syndromes are the same as those found in sporadic cancers. These include mutations in tumor suppressor genes such as TP53, BRCA1, and BRCA2, as well as DNA mismatch repair genes such as MLH1 and MSH2, and metabolic hub genes such as SDH (succinate dehydrogenase). The shared genetics of germline cancers and sporadic cancers have also helped to shed light on the key roles that specific types of somatic mutations play in sporadic cancers [16].

### 2.1. The Genetics of Familial Cancer

The existence of heritable cancers has led to two broader, largely unanswered questions in cancer genetics: (1) How heritable is cancer? and (2) How common is heritable cancer? Many sources have indicated that 5–10% of cancers are heritable [2,16]. This statement is often used by some authors to suggest that the heritability of cancer is also 5–10%. However, this estimate invariably appears without citations. It seems that this 5–10% estimate first appeared as a crude guess based on very limited breast cancer data from 1990 [17]. It has since been restated or reused in dozens of papers and websites. In other words, the 5–10% estimate for cancer heritability has become dogma through simple repetition. Such an estimate is likely not correct. It is also not very current nor is it applicable to other cancers. Clearly, more precise, and much more up-to-date estimates of the true heritability of cancer and the true prevalence of heritable cancer are needed, particularly if one is trying to understand cancer from a genetic or genomic perspective.

One way of updating this 5–10% estimate is to look more closely at the large cohort studies that have been undertaken over the past 30 years to determine both the heritability of cancer and the prevalence of heritable cancer. These include a number of large-scale twin cancer studies [18,19] and large-scale familial cancer studies [20]. The twin studies provided statistical estimates of the narrow-sense heritability of different cancers, while the familial studies provided estimates of the prevalence of heritable cancers [18,19] through analyzing family trees and assessing family-wise concordance in certain cancers [20]. These epidemiological/observational studies had a number of limitations (discussed later), but they did provide useful (upper limit) estimates of cancer heritability and heritable cancer prevalence. Alternate molecular measures of cancer heritability and heritable cancer prevalence are also available. For instance, with the advent of next generation sequencing (NGS), many large-scale surveys of known and suspected germline mutations in cancer patients have now been conducted. These surveys have provided even more exacting estimates of the true prevalence of heritable cancers [21,22,23,24,25,26,27,28,29,30,31,32,33,34,35,36,37,38,39]. Additionally, dozens of large-scale genome-wide association studies (GWAS) for many cancers have also been pursued. These GWA studies provided somewhat more precise, molecularly derived estimates of cancer heritability than twin studies [40,41,42,43,44,45,46,47,48,49,50,51]. By consolidating these studies and supplementing some of the missing twin heritability data from other studies [52,53,54], a summary these results has been prepared as shown in Table 1. Simply stated, Table 1 compares the prevalence of heritable cancers (in percentage terms) to the estimated heritability of cancers for the 12 most commonly diagnosed cancers in the USA (and most of the developed world). These 12 types of cancer account for ~78% of all known cancer cases in the US [55], and therefore, the numbers generated from this analysis can be reasonably extrapolated to all cancer types.

Several new and notable features are evident from Table 1. First, the prevalence of heritable cancers derived from NGS analysis of high penetrance germline mutations (Table 1, Column 3, Germline Prevalence) varies much more than the traditional “5–10%” that is often quoted [16,17], indeed, it ranges from a low of 0.3% in lung cancer to a high of 17.2% in prostate cancer. This serves to emphasize the fact that different cancers can have very different genetic contribution; some cancers, such as lung cancer, have almost none, and other cancers, such as prostate and breast cancer, have significant heritable contributions. The fact that some cancers have a range of prevalence values is simply a reflection of the genetic variability that exists in different ethnic populations. It also reflects differences in experimental design (populations size, mutational coverage, and chosen penetrance threshold). To simplify comparisons and calculations, an average prevalence value can be determined for those cancers where multiple germline prevalence values have been published. Using these average values, it is possible to determine that the case-weighted average prevalence of heritable cancers is 6.2%. In other words, the answer to Question #2 (How common is heritable cancer?) is: ~6%. Overall, this number indicates that germline/inherited cancers are relatively rare.

An alternative estimate of the prevalence of heritable cancer (or familial cancer) can be obtained from family cohort studies (Table 1, Column 4, Familial Prevalence). These studies, which measure the frequency of cancers among family members, provide an upper limit estimate to the prevalence or percentage of heritable cancers. Because they are observational or epidemiological in nature, family cohort studies do not provide the detailed molecular data found in germline NGS studies. Likewise, familial studies can be affected by shared environmental effects or other factors which artificially increase the estimated familial concordance rates for some cancers and lower them for others [20]. For instance, the authors of this study noted that the unusually high values for lung cancer (8.7%) were likely due to second-hand smoke exposure among family members and the high values for colorectal cancer (12.8%) were due to shared (cancer-inducing) diets [20]. Likewise, limited case numbers likely depressed the true prevalence values for kidney and liver cancer. Despite these caveats, and bearing in mind that these estimates correspond to the upper limit for heritable cancer, the observed range (2.6–20.2%) and the case-weighted average (10.2%) are reasonably close to those values seen in the NGS-derived germline prevalence column in Table 1. Taken together, these data suggest that, on average, 6–10% of cancers run in families.

The answer to Question #1 (How heritable is cancer?) is not quite as clear. The lower limit answer to this question is obviously 6.2% (the prevalence of heritable cancers we calculated above), but this lower limit estimate assumes that no other genes or no other SNPs contribute to the likelihood of developing cancer. However, it is known that many non-oncogenic genes exist that increase one’s risk for diabetes, obesity, alcoholism, Crohn’s disease, and other inflammatory or auto-immune diseases. These conditions are all also known to greatly increase the risk of developing cancer [56,57,58]. Interestingly, the prevalence of other family-wise diseases or co-morbidities also play a role in the prevalence statistics reported for family-wise measures of cancer. This is why they are regarded as upper limit estimates for heritable cancers. Therefore, it might be argued that the familial prevalence statistics in Table 1 or population attributable fractions for cancer could provide a reasonable estimate of cancer heritability [20,59]. Using this logic, it is possible to obtain a rough estimate of ~10% for the heritability of cancer.

A more robust estimate of cancer heritability can be obtained from GWAS studies on specific cancers. Hundreds of GWA studies have been performed on many different cancer types in an attempt to explore the issue of cancer heritability. Fortunately, many of these GWAS datasets have been made public [60]. It is also worth noting that cancer GWA studies have focused on higher-abundance SNP and copy number variants and ignored the rarer oncogenic germline mutations (such as BRCA1/2 or TP53). Therefore, the heritability estimates that GWA studies have provided are complementary to the heritability estimates provided by enumerating the prevalence of heritable cancers. In other words, by adding the GWAS heritability values to those measured for heritable cancer prevalence for each cancer type, it should be possible to obtain a more accurate estimate of the overall heritability of a given cancer. Using GWAS heritability data calculated and made available through the GWAS ROCS database [61], the heritability estimates for all 12 high-abundance cancers were tabulated, as shown in Table 1 (Column 5, GWAS Heritability). Note that, where multiple studies appeared in the GWAS ROCS database, only those with the largest subject numbers and/or highest number of significant SNPs were tabulated. As seen in Table 1, GWAS heritability estimates for many cancers (except breast and prostate) are quite low and range between 0.6 and 11.0%. Furthermore, the case-weighted average heritability for all 12 cancers is just 4.3%. Interestingly, for each of the 12 cancers, the sum of the germline prevalence and GWAS heritability values often come quite close to the family prevalence values (with the expected exceptions of lung cancer and colorectal cancer). Furthermore, the average GWAS heritability (4.3%) and the average germline prevalence (6.2%) give a summed heritability estimate of 10.5% for all cancers. This is almost exactly the same as the 10.2% heritability estimate derived for the family prevalence data. In other words, two separate lines of reasoning suggest that the average heritability of cancer is ~10%.

It should be noted that these two heritability estimates are quite different than the cancer heritability estimates derived via twin studies. Indeed, as seen in Table 1, (Column 6, Twin Heritability), most twin estimates are 3–40× larger than those derived from the GWAS or family prevalence estimates. In particular, the case-weighted average heritability for cancer via twin studies is more than 34%, while it is just 4.3% for GWAS studies, and just 10.2% for family studies. These findings give rise to the question “What is going on?”.

The short answer is that twin heritability estimates, especially for cancer, are flawed. A key assumption made in all twin studies is that monozygotic and dizygotic twins share a common environment [62]. This allows twin researchers to tease out the genetic influence while controlling for environmental effects. However, because cancer is a condition that typically develops in old age, long after twins have left their shared childhood homes and started independent lives, the assumption of a shared environment is fundamentally incorrect. Assuming a contribution for a shared environment that is too large will tend to greatly inflate any estimate of heritability [62]. More recently, sophisticated modeling methods using comprehensive genomic data have shown that twin heritability estimates for many conditions are consistently 2–3× higher than those determined via molecular methods [63,64]. Likewise, because twins are rare occurrences and twins with shared rare conditions are even rarer, the influence of small data samples tends to inflate twin heritability estimates or leads to enormously large standard errors or unreasonably wide confidence intervals. This phenomenon has been highlighted by the remarkably discordant measurements and widely publicized reports on the heritability of autism [65,66]. Finally, most of the twin heritability estimates shown in Table 1 do not align with the known causes of certain cancers. For instance, more than 95% of lung cancers can be explained through known, excess exposures to tobacco smoke, pollutants, radon, asbestos, or lung infections [56,67]. This is obviously inconsistent with lung cancer having a heritability of 18% [18]. Likewise, more than 90% of melanomas are known to be caused by excess UV radiation exposure [68]. This fact is obviously inconsistent with melanoma having a heritability of 53% [18]. Nevertheless, the heritability estimates derived from Table 1 via the germline prevalence + GWAS heritability data (1.6% for lung cancer and 3.4% for melanoma) seem to be much more in line with the known epidemiology of these cancers.

Unfortunately, as flawed as twin estimates of cancer heritability are, they are widely viewed as absolute truths within the cancer genomics community [18,62,63,64,65,66]. Indeed, the phenomenon of repeating a falsehood sufficiently often to make it appear true, seems to be at work once again. The belief in twin studies is so great that considerable work continues to be directed within the statistical genomics community to develop ever more sophisticated models to ensure GWAS data or NGS data on heritability fits with observed twin data on heritability [63,64], rather than the other way around. It will be important for the cancer genomics community to move beyond this infatuation with twin studies and to embrace a much more reasonable and reasoned view about the heritability of cancer. Indeed, as shown here (and elsewhere) the weight of evidence suggests that the heritability of cancer is ~10% and that this heritability ranges from a low of 1.6% for lung cancer to a high of 21.1% for prostate cancer. These lower heritability estimates are much more aligned with the abundant evidence that cancer is more an environmental disease and less an inherited disease [56].

### 2.2. The Genetics of Sporadic Cancer

While most of our focus has been on estimating cancer heritability and the prevalence of inherited cancer, this exercise served to highlight the fact that >90% of cancers are sporadic, that is, they have no germline origins. However, this does not mean that genetics does not play a role in the development of sporadic cancers. Indeed, most sporadic cancer cells do have mutations. These mutations are not inborn or inherited, but rather they are acquired. Of course, a key question that genetics does not answer is “How are these somatic cancer-causing mutations acquired?”. According to the somatic mutation theory (SMT) [8], these acquired mutations arise from external factors (mutagens) or environmental exposures leading to genetic instability. These initiators of cancer, which are arguably more important than the genetic lesions themselves, are discussed later in this review.

When tumors (sporadic or inherited) are sequenced, it is not unusual to see large numbers of mutations. Typically, any given tumor genome will exhibit 100–150 protein altering mutations, of which 10–12 are so-called “driver” mutations, and the remaining are called “passenger” mutations [69,70]. The driver mutations promote or drive carcinogenesis, while the passenger mutations are simply incidental, arising from the genomic instability that is inherent with many tumors. Different cancers will tend to exhibit different numbers of cancer driver genes with some, such as certain types of kidney cancer, having as few as two and others, such as endometrial cancer, having as many as 55 [70]. Nearly 300 cancer driver genes have been identified to date [70], all of which can be tied to the 587 known cancer-associated genes in humans documented in the COSMIC database [71]. Importantly, the ability to identify specific driver genes in tumors has opened the door to precision oncology, whereby, targeted therapies may be used to interfere with key driver mutations in specific tumor types [70].

Most cancer-associated or cancer driver genes fall into two broad categories: (1) tumor suppressor genes and (2) oncogenes or proto-oncogenes [1]. Tumor suppressor genes act to suppress cell proliferation and tumor development. In other words, they are anti-oncogenes. If a tumor suppressor gene is mutated, damaged, or altered epigenetically, it can lead to cell proliferation and oncogenesis [1]. Tumor suppressors fall into six main categories: (a) cell cycle control genes or cell division inhibitors, (b) hormone or growth factor receptors, (c) checkpoint control genes, (d) apoptosis inducers, (e) cell adhesion genes, and (f) DNA repair genes. In contrast to tumor suppressor genes (which act as carcinogenic brakes), oncogenes act as carcinogenic accelerators. More specifically, oncogenes are mutated genes that contribute to the development of a cancer [1]. Unmutated oncogenes are called proto-oncogenes. Oncogenes fall into five categories: (a) growth factors, (b) growth factor receptors, (c) signal transducers, (d) transcription factors, and (e) programmed cell death regulators. As shown in the COSMIC database [71] and as highlighted by the functional categories given above, tumor suppressors and oncogenes appear to play roles in just six of the ten hallmarks of cancer [11]. These include: (1) inducing genome instability, (2) sustaining proliferative signaling, (3) evading growth suppressors, (4) resisting cell death, (5) maintaining replicative immortality, and (6) activating invasion and metastasis. Notably, oncogenes and tumor suppressors appear to play little or no role in at least four other cancer hallmarks including angiogenesis, metabolic dysfunction, evading immune destruction, and tumor-promoting inflammation [1,11]. This suggests that other, non-genetic factors must also play roles in initiating and sustaining tumor growth [12,13]. Furthermore, since it is widely believed that cells typically need two or more driver mutations in cancer-associated genes to become carcinogenic [72] and because these genes typically have one function each, this implies that nascent tumors may only exhibit two of the ten known cancer hallmarks. This is also somewhat problematic as small tumors typically exhibit most, if not all, of the ten hallmarks of cancer [11]. In addition, some tumor types exhibit very large numbers (>40) of driver mutations, which begs the question “How can so many independent driver mutations be acquired in a single tissue?”. Again, this suggests that other, non-genetic factors must play roles in both initiating and sustaining tumor growth [12,13]. These non-genetic contributions to tumorigenesis are discussed in more detail below.

## 3. Cancer as an Environmental Disease (the Exposome View)

Regardless of whether one is looking at sporadic cancer or germline/familial cancer, it is important to remember that almost no one is born with cancer. Cancer is an acquired disease [2,56]. It typically takes years or decades to develop or manifest. Indeed, most cases of familial cancer are only detected in a person’s third or fourth decade, while most cases of sporadic cancer appear after a person’s sixth decade [2,16,20]. Even among those individuals with germline mutations (giving them a strong innate propensity towards cancer), other insults, injuries, infections or mutations within specific tissues must occur to initiate cancer. For those who develop sporadic cancer, the same initiating or mutagenic events in somatic cells must occur, but “lightning must strike twice” in the same tissue or organ to initiate cancer [72]. The fact that cancer is an acquired disease, initiated through decades of chronic exposures, is often forgotten by those who view cancer purely through a genetic lens. However, thanks to the work of cancer epidemiologists and agencies such as the International Agency for Research on Cancer (IARC), the role of the environment and importance of the exposome in cancer initiation and development can no longer be ignored.

The concept of the exposome was first introduced by Dr. Chris Wild, a past director of IARC, in 2005 [73]. While the definition of the exposome has gone through several iterations, it is now formally defined as the measure of all the exposures (including lifestyle factors) of an individual in a lifetime and how those exposures relate to health. These exposures include environmental factors such as chemical contaminants (exogenous small molecules), radiation, food, tobacco smoke, pollutants as well as lifestyle factors such as physical activity, stress, occupation, education, quality of housing, and climate. Dr. Wild was certainly not the first person to note the effect of occupational or environmental exposures on cancer incidence and prevalence. Chimney sweep cancer (a form of scrotal skin cancer) was first noted in 1775 among London’s chimney sweepers who were chronically exposed to soot [3,4]. More than a century passed before other environmental causes of cancer were detected or described. Radiation-induced cancer was first noted in 1902 [74], while the first report of specific chemicals (coal tar) inducing cancer was shown in 1915 [3,4]. The link between tobacco smoke and cancer was made in 1923 [3], while the connection between viruses and cancer was shown in 1926 [5]. Interestingly, it was not until 1940 that the relationship between diet, nutrition/lifestyle, and cancer was first elucidated [75]. All of these relationships underline the importance of the environment in both initiating cancer and in reprogramming the DNA within cells to sustain cancer growth. It was because of these many discoveries concerning known and suspected carcinogens, exogenous chemicals, and cancer-causing lifestyles that IARC started publishing expert-reviewed monographs on carcinogenic agents in 1970. These monographs, along with other IARC databases such as Exposome Explorer [76,77], highlight the close link between the exposome and cancer.

Currently, IARC categorizes carcinogenic agents or carcinogenic exposures into four categories: (1) Group 1 (definitely carcinogenic), (2) Group 2A (probably carcinogenic), (3) Group 2B (possibly carcinogenic) and (4) Group 3 (not classifiable or not carcinogenic). Among the Group 1 agents, IARC has identified 11 pathogens (virsues, bacteria, and parasitic worms), 54 chemicals, 15 radiation sources (including radioactive chemicals), and 48 mixture exposures or exposure circumstances (consisting of foods, drugs, household, or occupational exposures). Among the group 2A agents, IARC lists 67 chemicals, 3 pathogens, and 14 mixture exposures or exposure circumstances. While many of the IARC-identified agents are known mutagens (causing DNA mutations or chromosomal damage), many carcinogenic agents are not. These include a number of chronic inflammatory agents.

For instance, with the exception of human papilloma virus, most of the known cancer-associated pathogens identified by IARC are not genetically integrative. Rather, they appear to cause cancer through chronic inflammation, immunosuppression, or irritation [78,79,80]. Of course, chronic inflammation can induce cell proliferation and can generate free radicals as well as N-nitroso compounds that are potentially mutagenic. However, inflammation can also induce other metabolic, immunosuppressive, and epigenetic changes that are also key to cancer progression. Other food or lifestyle carcinogens identified by IARC such as red meat, wood dust, hot beverages, night shift work, oral contraceptives, and post-menopausal estrogen therapy appear to cause cancer through inflammation, irritation, disrupted circadian rhythm, or chemical activation of cell growth pathways [81,82,83].

While the list of known carcinogens and mutagens identified by IARC is quite large, it is important to note that the IARC list does not include many other dietary, lifestyle, or chronic disease contributions to cancer. For instance, diabetes, obesity, chronic inflammatory diseases, lack of physical activity, mineral and vitamin deficiencies, and poor diet are not mentioned in the IARC list, yet they are all known to greatly increase the risk of cancer [56]. For these kinds of exposures, it is believed that oxidative stress, reactive oxygen species, irritation, inflammation, and immunosuppression seem to be the main drivers of carcinogenesis [56,84]. Oxidative stress and inflammation can lead to somatic mutations which likely arose from dysregulated DNA repair [84]. However, oxidative stress and inflammation also appear to cause metabolic and mitochondrial dysfunction, which triggers additional cancer-like activity and cancer-like hallmarks within susceptible cells [13].

Given the plethora of known mutagens and carcinogens along with the growing list of dietary, lifestyle, and chronic diseases associated with increased cancer risk, it is perhaps appropriate to ask “What portion of cancers are caused (either directly or indirectly) by the environment?”. Some estimates suggest it is as high as 90–95% [56], others suggest it is closer to 42–43% [85], with a large portion left to “unknown causes”. Certainly, from our earlier estimates of the heritable proportion of cancer (6–10%), one could argue that an estimate of 90–94% for the environmental contribution to cancer would seem reasonable.

However, this cancer-wide estimate does not provide a number for the individual contributions of these exposures. For instance, “What proportion of cancer is due to smoking?”, “What proportion is due to infectious agents?”, and “What proportion is due to chemical exposures?”. Unfortunately, the specific contribution of these individual environmental agents to cancer are rather difficult to find. One very approximate estimate, from 2008, suggested that diet and alcohol contributed to 35–40% of observed cancer cases, tobacco contributed 25–30%, infections contributed 15–20%, obesity contributed 10–20%, and other environmental exposures contributed 10–15%. However, these estimates relied on rather dated statistics and simplified models. They also significantly overestimated the influence of diet and infections on cancer incidence (especially in the USA), while ignoring other major contributions to cancer incidence (such as radon exposure, asbestos, diabetes, outdoor pollution, etc.). A more recent estimate of cancer suggests that smoking contributed to 19% of cancer cases, obesity contributed to 8%, diet and alcohol contributed 10%, infections contributed 3.4%, and other causes contributed 7% [85]. However, this estimate suggests nearly 60% of cancers have either an unknown or a purely genetic cause. Furthermore, this 2018 estimate also neglects other well-known environmental causes of cancer (such as radon exposure, asbestos, diabetes, outdoor pollution, etc.).

Using more recent literature, more complete estimates, and focusing on known causes of cancer mortality, which tend to be more accurately reported, it is possible to come up with a more precise estimate of the individual environmental (and genetic) contributions to cancer and, more specifically, cancer mortality [85,86,87,88,89,90,91,92,93,94,95,96,97,98,99,100,101,102,103,104,105,106]. These results are shown in Table 2.

In Table 2, there are several points to note regarding the risk factors and causes of cancer deaths. First, the percentage of heritable/germline cancer deaths was calculated using the fraction of heritable cancer cases listed in Table 1 scaled with the cancer mortality data in [86]. This produced a number that is smaller than the total percentage of heritable cancer cases (3–6% instead of 6–10%). Second, it is important to note that the diagnosis of cancer actually leads to a surprisingly large number of iatrogenic, adverse drug responses, or medical error deaths [96]. This was estimated from the overall proportion of reported iatrogenic deaths in the USA [96]. This iatrogenic component represents an undercounted and unappreciated effect of the “exposome” on cancer mortality. Third, while the data in Table 2 were compiled using U.S. data, the distribution with regard to the causes of cancer deaths is likely very similar for most countries in the developed world (Canada, Australia, Japan, most of Europe). In the developing world, the fraction of cancer deaths due to infectious organisms is much higher (12–16% vs. 4–5%) [91] while the faction of cancer deaths due to meat consumption, diabetes, and obesity is typically much lower (4–5% vs. 12–15%). However, these differences still lead to similar total fractions of explainable cancer deaths (i.e., 75–80%). They also show that tobacco smoke continues to be the leading cause of cancer deaths both in the developed and developing world.

There are at least two takeaway messages that should be obtained from Table 2. The first is that 75–80% of all cancer deaths in the USA can be explained through either exposome effects (~70–75%) or heritable effects (~5%). In other words, the proportion of cancer deaths, and by inference, the proportion of explainable cancer causes, is much higher than previously reported [85]. The second takeaway message is that cancer is fundamentally a disease of aging [107]. As seen in the top line of Table 1, more than 70% of cancer deaths occur in people >65. This fact underlines the point that cancer is a disease that is acquired through decades of incidental, often innocuous exposures. Simply breathing (which produces reactive oxygen species and oxidative stress), eating or drinking (which has similar stressful effects on the body), or managing day-to-day stress wears down the body. At a cellular level, these erosive effects lead to mutations, chronic inflammation, reduced immune response (allowing cancer cells to escape detection), mitochondrial dysfunction, and ultimately cancer [107]. This would strongly suggest that a significant portion of the missing 20% (in Table 2), in terms of explainable causes of cancer, is probably associated with the simple effects of aging. Indeed, if anyone lives long enough, they are almost certain to develop at least one form of cancer [2,107]. In addition to the effects of aging, it is also likely that the effects of the listed exposures or lifestyle choices on cancer incidence and cancer mortality, especially diet and physical activity, are underestimated [56,85]. As more is learned about their real effects on cancer incidence and mortality, it is expected that their population attributable fraction will rise by perhaps another 5–10%. Other contributions to cancer incidence and mortality, such as drug use/abuse [108], chronic disease [109], increased height [110], and gut microbiome effects [111] were not included in Table 2, as their effects on cancer cases or mortality have not been fully enumerated. However, as more data are acquired about these effects, it is reasonable to assume that, when combined together, they will contribute another 5–10% to the explainable portion of cancer cases and cancer mortality.

**Figure 1:**
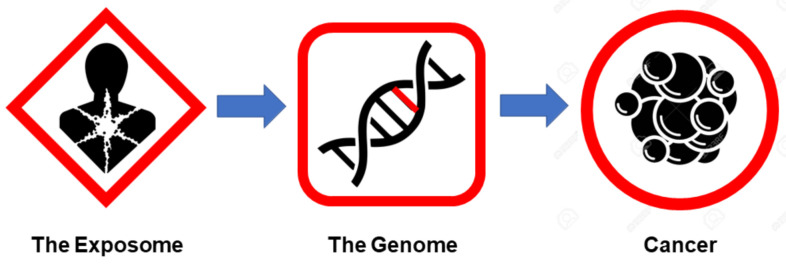
A simplified depiction of how the somatic mutation theory (SMT) and the genomic view of cancer explain oncogenesis. Environmental exposures (the exposome) lead to mutations in the genome which lead to tumor development.

As shown in Table 2 and highlighted throughout this section, from the perspective of the exposome, cancer is not a genetic disease nor is it a genetically inevitable disease. Rather, cancer is an acquired disease that can largely be prevented [56]. Indeed, most of the significant improvements seen in reduced cancer incidence and mortality over the past two to three decades have been through advances in cancer prevention and cancer screening rather than in gene-guided precision cancer treatments [55,112,113]. From an etiological perspective, the exposome basically explains how most cancers are initiated. That is, certain environmental or lifestyle agents appear to cause the mutations, genetic instability, inflammation, or reduced immune surveillance needed to start carcinogenesis. Once initiated, the genetic or epigenetic changes caused by the exposome are propagated to other cells via the newly altered genome. This cell-mediated, genetically driven propagation ultimately leads to the appearance of detectable tumors or detectable cancerous tissues. This linear process of the exposome leading to mutations in the genome, which in turn lead to cancer (Figure 1), is fundamental to the somatic mutation theory (SMT) of cancer [8].

However, this is still an incomplete picture of how cancer is initiated, propagated and sustained. Indeed, the influence of the exposome only explains one aspect (initiation) and just one hallmark of cancer (genetic instability). Additionally, the influence of the genome (i.e., driver genes) only appears to explain the propagation aspect of cancer and perhaps four or five cancer hallmarks. For a more complete understanding of how cancer is sustained and how the other hallmarks of cancer can be explained, we need to look at cancer from the perspective of the metabolome.

## 4. Cancer as a Metabolic Disease (the Metabolome View)

Prior to 1970, most cancer researchers thought of cancer as a metabolic disorder. In 1927, Otto Warburg noticed that cancer cells exhibited a distinct metabolic phenotype, consuming 200× more glucose than normal cells (the “Warburg effect”) [6]. This metabolic dysregulation was marked by a shift away from mitochondrial-based oxidative phosphorylation (OXPHOS) toward cytoplasmically driven aerobic glycolysis. In other words, cancer cells moved from being inert entities producing large amounts of ATP (a fundamental characteristic of OXPHOS) to rapidly dividing cellular engines generating huge quantities of amino acids, nucleotides, and fatty acids needed to produce the biomass (proteins, lipids, and DNA/RNA) for sustained cellular growth and division. Warburg’s discovery was noted by many other investigators, and it explained not only the dysregulated metabolism seen in cancer, but it also explained how cancer cells could replicate forever, how they could resist cell death, how their proliferation signals could persist, and how they could avoid growth suppression (i.e., five of the 10 hallmarks of cancer [11]).

Indeed, because Warburg’s findings cast such an enormous influence over the cancer community, most cancer drugs discovered in the 1950s and 1960s were called “antimetabolites” [114]. Strictly speaking, an antimetabolite is a metabolite-like compound designed to interfere with cellular metabolism, especially with DNA synthesis. Indeed, antimetabolites such as 5-fluorouracil, methotrexate, and 6-mercaptopurine are still widely used today and serve as very effective cancer therapies. However, Warburg’s theory of metabolic dysregulation did not really explain how cancer was initiated or acquired. Likewise, it did not explain how cancer cells could be propagated through multiple generations or why so many genetic instabilities/mutations were found in cancer cells. With Warburg’s death in 1970 and the discovery of oncogenes in the same year [2], most cancer researchers rapidly shifted their thinking to view cancer as almost exclusively a genetic disease rather than a metabolic disease. This genetic/genomic perspective has largely come to dominate today’s thinking about cancer, ranging from its origins and etiology to its diagnosis and treatment.

The “re-discovery” of cancer as a metabolic disorder has mostly occurred in the last 10–15 years. This shift in thinking is primarily due to the increased awareness and accessibility of metabolomics [115]. This, in turn, has led to the realization that many oncogenes and tumor suppressors actually serve as metabolic hubs [116,117]. For instance, oncogenes such as PI3K/Akt and BCR-ABL are both known to enhance glucose uptake and increase hexokinase II activity [117]. Likewise, activation of the c-Myc, Ras, and Her2/Neu oncogenes leads to enhanced glycolysis [117,118]. In contrast, the tumor suppressor p53 promotes oxidative phosphorylation, while its loss leads to glycolysis. Similarly, the tumor suppressors SDH (succinate dehydrogenase), FH (fumarate hydratase), and IDH (isocitrate dehydrogenase) are responsible for maintaining the tricarboxylic acid (TCA) cycle, while their loss leads to genetic instabilities and epigenetic alterations [117]. This connection between oncogenes and metabolism has brought the genomic view of cancer into closer alignment with the metabolomic view. In addition to building bridges between the genetic and metabolic perspectives on cancer, metabolomics has also led to three other important advances: the delineation of key metabolic pathways or metabotypes involved in cancer [14,117], the discovery of “oncometabolites” [10], and the elucidation of how cancer-associated metabolites and cancer-associated metabolism explain many of the hallmarks of cancer [119].

The identification of the key cancer metabolic pathways or metabotypes represents one of the more significant contributions of metabolomics to the field of cancer research. Essentially, almost all cancers exhibit one or more forms of metabolic dysregulation [14,119]. These include: (1) aerobic glycolysis, (2) glutaminolysis, (3) disrupted one-carbon metabolism, and/or 4) altered metabolism of essential amino acids [14,117]. Aerobic glycolysis, a metabolic process found in many proliferating cells, is characterized by high levels of glucose consumption, modest energy production, significant lactate production, and the generation of the nucleotides and lipid precursors needed for cell biosynthesis [120]. The genes involved in glycolysis are overexpressed in 70% of known cancers, with lymph node, prostate, kidney, and brain cancer exhibiting particularly high levels of expression of these glycolytic genes [121,122]. Aerobic glycolysis is sometimes called “glucose addiction” as it highlights the critical need for glucose to sustain cancer cell growth. Glutaminolysis or “glutamine addition” is another type of metabolic process found in actively proliferating cells. It is characterized by unusually high levels of glutamine uptake. Glutamine is the most abundant amino acid in blood and is an important source of energy for many tissues. In glutaminolysis, the amino acid glutamine is broken down and converted to TCA intermediates and other nitrogen-containing precursors that can, then, be used to produce nucleic acids, certain amino acids, and lipids. Glutaminolysis is also essential for maintaining redox homeostasis, via the production of glutathione, making cancer cells more tolerant to reactive oxygen species (ROS) [123]. Glutamine and glutaminolysis play key roles in cancer cell growth signaling (via the mTOR pathway), providing biomass, energy, and antioxidants to help cancer cells replicate continuously, avoiding growth suppression by reducing autophagy, limiting cell death, and activating cellular invasion or metastasis. Glutaminolysis is commonly seen in lung, breast, bladder, and blood cancers, as well as other c-Myc-driven cancers [118,124].

In addition to aerobic glycolysis and glutaminolysis, several cancers, particularly breast and lung cancer, lymphoblastic leukemia, neuroblastoma, and melanoma, exhibit dysregulation of one-carbon metabolism [125]. One-carbon metabolism involves the use of methionine, glycine, serine, choline, and folate as sources of methyl groups needed for the synthesis of DNA, polyamines, amino acids, creatine, and phospholipids. These methyl groups are also essential for the methylation of histones and DNA. Therefore, dysregulated one-carbon metabolism is believed to contribute to the epigenetic changes often seen in cancer [125]. A less appreciated form of metabolic dysregulation in cancer lies in the use (or misuse) of essential amino acids by cancer cells. Essential amino acids must be obtained from the diet, but if insufficient quantities are available, the body will often scavenge these amino acids from muscle tissue [126,127]. This metabolic scavenging, which can arise from both tumor growth and tumor-induced inflammation, gives rise to cancer cachexia (muscle wasting), which is particularly common in pancreatic, gastric, lung, esophageal, colorectal, as well as head and neck cancer [127,128]. Essential amino acids, especially branched chain amino acids, and their breakdown products (such as kynurenine and polyamines) are also used as signaling molecules to increase anabolic processes (via mTOR), induce inflammation, support immunosuppression, or enhance cellular proliferation—all of which are key hallmarks of cancer [129].

The fact that there may be just four major metabolic pathways or metabotypes associated with cancer has been something of a revelation. Rather than viewing cancer as an incredibly complex genetic disorder with each tumor being one of a combinatorial infinite collection of dozens of different oncogenic mutations, it is now possible to look at cancer as being a far more finitely defined disease [14]. Indeed, it appears that most cancers appear to be classifiable into a small (<10) number of unique metabotypes or combinations of metabotypes. This opens up some interesting opportunities with regard to diagnosing and even treating cancer [14,119].

The other key contribution of metabolomics to our understanding of cancer has been the discovery of oncometabolites. Oncometabolites are endogenous metabolites whose accumulation initiates or sustains tumor growth and metastasis [10,14,130]. The first oncometabolite to be discovered was 2-hydroxyglutarate (2-HG), a relatively rare metabolite that is found in high concentrations in gliomas [10]; 2-HG (especially the D-isomer) inhibits histone lysine demethylases leading to altered histone methylation patterns. This activates oncogenes and inactivate tumor-suppressor genes, ultimately leading to carcinogenesis. Since 2-HG’s discovery in 2009, two additional widely recognized oncometabolites have been identified, i.e., fumarate and succinate. These compounds also induce genetic and epigenetic changes through a similar mechanism, leading to carcinogenesis [130]. However, the “requirement” that an oncometabolite must be inherently mutagenic or that it must physically alter the genome is incorrect. Indeed, such a view tends to ignore the many other roles that oncometabolites play in carcinogenesis. For instance, 2-HG not only leads to genome instability, but it also induces angiogenesis [131], prevents apoptosis or necroptosis [132], leads to immunosuppression [133], and actively signals cell growth via the mTOR pathway [134]. By considering the broader roles that oncometabolites (and other endogenous metabolites) play in carcinogenesis and by remembering that oncometabolites are simply metabolites that initiate or sustain tumor growth, then, it is possible to identify many more oncometabolites than commonly acknowledged. Table 3 provides a reasonably complete list of oncometabolites assembled from the current literature [135,136,137,138,139,140,141,142,143,144,145,146,147,148,149,150,151,152,153,154,155,156,157,158,159,160,161,162,163]. This list includes a number of new or lesser known oncometabolites that have been recently identified, as well as a number of well-known metabolites that play key roles in oncogenesis, but which have not been “officially” identified as oncometabolites.

Table 3 provides information about different oncometabolites and about the cancers with which they are associated, the mechanisms by which these oncometabolites work, along with the hallmarks of cancer [11] to which they contribute. As seen from this table, there are several broad categories to which oncometabolites belong. A number (fumarate, succinate, and 2-HG) are TCA intermediates, some are amino acids (glutamine, asparagine, glycine, leucine, isoleucine, lysine, etc.), others are polyamines (spermine and spermidine), while still others are hormones or bile acids (estrogen, progesterone, and lithocholic acid). It is also evident that certain oncometabolites are quite specific to certain types of cancers, while others (such as homocysteine, glucose, and lactate) are found in almost all cancers.

It is important to note that different oncometabolites work through different mechanisms. As shown in Table 3, some oncometabolites only appear to a have a small number of oncogenic functions (SAICAR, uric acid, and succinylacetone), while others such as 2-HG, glutamine, and arginine have a much larger number of oncogenic functions or roles. Many of these oncometabolites are known cancer biomarkers, with radioactive derivatives of glucose and glutamine being widely used in PET-based tumor imaging [164]. Other oncometabolites are much more cancer-specific and may be elevated in blood or urine depending on which type of cancer is manifested [165,166,167,168,169,170]. What is most striking about the data in Table 3 is how many of the hallmarks of cancer can be explained by this relatively short list of 38 oncometabolites. Collectively, every one of the 10 known cancer hallmarks [11] can be rationalized by at least one of these oncometabolites. In fact, just three nearly ubiquitous oncometabolites (lactate, glutamine, and glucose) can explain or participate in processes that explain all 10 cancer hallmarks. Much more detailed information regarding how many of these oncometabolites play roles in manifesting or rationalizing the hallmarks of cancer has been provided in several recent reviews [13,14,117,119].

Interestingly, the coverage or “explainability” of all 10 cancer hallmarks by this set of 38 oncometabolites (or even just the oncometabolites lactate, glutamine, and glucose) is somewhat more than the six hallmarks of cancer that can be explained by the nearly 600 known oncogenes and tumor suppressors. This numeric discrepancy underlines the importance of metabolites and metabolism in cancer. Indeed, as seen in Table 3, oncometabolites play a key role in not only initiating but in sustaining cancer. This metabolic sustenance often leads to further genetic instability, leading to even greater metabolic dysregulation. This more expansive view of what constitutes an oncometabolite certainly helps us understand why oncometabolites are so important and why metabolic dysregulation, and the consequences that arise from it, can explain so many of the hallmarks of cancer. Clearly, as the definition of what constitutes an oncogene or tumor suppressor continues to expand (i.e., through the inclusion of more metabolic enzymes and transporters), it is likely that the ability of these genetic cancer drivers to explain the hallmarks of cancer will also expand.

## 5. Connecting the Multiple Views of Cancer through Metabolomics

As show throughout this review, the genome, the exposome, and the metabolome all play roles in the development and manifestation of cancer. The vast majority of cancers are initiated by environmental exposures (the exposome) which lead to cancer-inducing genetic changes. The resulting genetic changes are, then, propagated through the altered DNA of the proliferating cancer cells (the genome). Finally, the dividing cancer cells are nourished and sustained by genetically reprogrammed, cancer-specific metabolism (the metabolome). This multi-step view of carcinogenesis, where the exposome initiates it, the genome propagates it, and the metabolome sustains it, certainly provides a more holistic, multi-omics view of the disease. It also helps explain how each of these different “omes” contribute to the hallmarks of cancer. However, it will be shown later that even this linear, multi-omics view is incomplete. Furthermore, such a simplified perspective does not fully explain how the individual components within these different cancer “omes” ultimately lead to cancer or how they underlie each of the hallmarks that characterize cancer. Therefore, a key challenge over the past two decades has been trying to identify the constituents that define the cancer genome, the cancer exposome, and the cancer metabolome.

Thanks to metabolomics, it has been possible to identify many of these omic constituents and to explore how the genome, exposome, and metabolome interact to initiate, propagate, and sustain cancer. For instance, the study and characterization of the cancer exposome has been made much easier by advances in metabolomics. Nearly all the 200+ organic compounds in the IARC lists of known or suspected carcinogens can be identified, quantified, or monitored via mass spectrometry (MS)-based metabolomic methods [171,172,173,174,175,176]. With increased accessibility to metabolomics resources, it is now fairly routine to measure these compounds in biofluids, tissues, and the environment [177,178]. Likewise, most of the 50+ inorganic, metal, or mineral carcinogens in the IARC lists can be detected, quantified, or monitored via metabolomic methods, especially those that use inductively coupled plasma (ICP) mass spectrometry [179,180]. Similarly, many of the dietary or lifestyle exposures identified by IARC or highlighted in Table 2 of this paper can also be detected, either directly or indirectly, via metabolomic methods [181,182,183,184,185]. More importantly, the molecular effects and the molecular consequences of these exposures on cells, tissues, or biofluids can also be characterized via metabolomics [186,187,188,189]. While the genetic consequences of different exposures can also be detected via genomics or transcriptomics, it is important to note that metabolomics provides more useful insights into the inflammatory, immunosuppressive, signaling, and metabolic changes which more directly affect a cancer’s progression or phenotype [178,190].

Technical and methodological advances in metabolomics have also led to a new understanding of the cancer metabolome. Indeed, the application of metabolomics to cancer research has led to the discovery of dozens of oncometabolites [135,136,137,138,139,140,141,142,143,144,145,146,147,148,149,150,151,152,153,154,155,156,157,158,159,160,161,162,163] and literally hundreds of metabolite-based cancer biomarkers [165,166,167,168,169,170], some of which are already being used in clinics [164,191,192]. Furthermore, metabolomics has enabled the identification of a number of key cancer metabotypes (or metabolic phenotypes), which has revealed that a relatively small number of key metabolites and an even smaller number of metabolic processes contribute significantly to the hallmarks of cancer [14,117,119]. These metabolic discoveries are leading to a better understanding of the molecular mechanisms underlying carcinogenesis, and to a better understanding of how to treat cancer.

In particular, the observation that certain endogenously produced metabolites (i.e., oncometabolites) can cause cancer certainly suggests that their depletion or reduction could potentially arrest cancer. For instance, depleting dietary glucose (a key oncometabolite) through low carbohydrate, ketogenic diets has been shown to have positive effects in patient survival, tumor sensitization, and tumor shrinkage [13,193]. Similarly, caloric restriction, intermittent fasting, or the deprivation of certain “oncogenic” amino acids in the diet has also been shown to have positive effects in cancer for various animal models and in some cancer patients [194,195,196,197]. Likewise, the addition of certain basic compounds (such as bicarbonate, lysine, or Tris) in the diet to buffer against the effects of lactate and the general tumor acidosis has shown some unexpected benefits in both animal tumor models and in some cancer patients [198,199,200]. In many cases, the anticancer effects of these dietary modifications were amplified with the inclusion of more conventional chemotherapies or antimetabolite therapies [196,200].

Metabolomics has also enabled the identification and mechanistic characterization of metabolites or dietary compounds that are anti-oncometabolites or cancer preventing agents. For instance, metabolome-wide association studies have shown that individuals with high plasma levels of vitamin C, carotenoids, and alpha-tocopherol were protected against gastric cancer [201], while those with high calcium and vitamin D levels were protected against colorectal cancer [201]. More recent studies have shown that higher plasma levels of valine, leucine, and bilirubin also protect against colorectal cancer [202]. Whether these are simply associations or whether these metabolites have a true cancer protective or anti-oncogenic effect still needs further work. However, the anti-oncogenic effect of endogenously produced short-chain fatty acids (SCFAs) is much clearer. Once again, metabolomics has also played a key role in characterizing SCFAs and their antitumor effects, especially in colorectal cancer [203,204]. As shown through a number of studies, SCFAs, such as butyric acid, acetic acid, and propionic acid, can act as histone deacetylase inhibitors or as autophagy/apoptosis promoters [204,205]. The production of SCFAs is largely driven by gut microbiota which convert dietary fiber into these anti-oncogenic fatty acids. This largely explains why those with high levels of dietary fiber have much lower levels of colorectal cancer [201].

As the role of metabolites as genetic signaling molecules or as the products of specific (mutated) metabolic enzymes has become clearer, so too has the connection between the metabolome and the genome been strengthened. Thanks to metabolomics, more and more metabolic enzymes are being listed as oncogenes and tumor suppressors [116,206]. Likewise, many cancer-associated genes are being re-evaluated for their roles as metabolic hubs or as components in metabolic pathways, largely through metabolomic studies [118,207,208,209]. The fact that metabolites can serve as substrates for genome-encoded enzymes and the fact that metabolites can both activate or suppress the activity of genes and proteins has also made metabolites (or antimetabolites) more interesting to genome-oriented cancer researchers. Historically, some of the most successful cancer chemotherapies (5-fluorouracil, methotrexate, and 6-mercaptopurine) have targeted enzymes associated with nucleotide synthesis, such as thymidylate synthase or hypoxanthine-guanine phosphoribosyltransferase. More recently, cancer chemotherapies have begun to target other kinds of biosynthetic enzymes such as the tumor suppressor isocitrate dehydrogenase (IDH). IDH was first identified as a tumor suppressor because its loss of function led to the production of the oncometabolite 2-HG [10]. Metabolomic studies helped reveal the crucial connection between IDH function and 2-HG production and this led to the development of at least two IDH-targeting drugs. One was developed for IDH2 inhibition (enasidenib) and the other for IDH1 inhibition (ivosidenib); both are now approved by the FDA [210,211]. These IDH inhibitors are now used to treat acute myeloid leukemia, while ivosidenib is also being used to treat cholangiocarcinoma. The success of drugs that alter cancer metabolism and the cancer metabolome has led to explorations into re-purposing other metabolism-altering drugs to serve as anticancer therapies. Drugs such as metformin (a diabetic biguanide that inhibits hexokinase II), dichloroacetate (a lactic acidosis drug that inhibits pyruvate dehydrogenase kinase), orlistat (an anti-obesity drug that blocks fatty acid synthase), and statins (anti-cholesterol drugs that inhibit 3-hydroxy-3-methylglutaryl-coenzyme A reductase) are all showing promise as anticancer therapies or cancer prevention prophylactics [119,212]. These findings suggest that cancer, if viewed primarily as a metabolic disorder, may be somewhat simpler to treat and simpler to understand than if it is viewed primarily as a genetic disorder [14].

## 6. The Big Picture View of Cancer

On the one hand, from a genetic view, cancer can seem impossibly complex, with each tumor exhibiting almost innumerable genetic faults and variations. On the other hand, from a metabolic perspective, cancer appears to be a relatively simple disease characterized by a remarkably small number of distinct metabolic phenotypes. When viewed from this metabolic perspective, the role of both primary and secondary metabolites in carcinogenesis also becomes clearer. In particular, rather than serving merely as nutrients or building blocks, (onco)metabolites function as important cellular regulators and cellular signaling molecules helping to initiate and sustain carcinogenesis. Indeed, when it comes to cancer, metabolites may play a role that is equal to or even exceed that often ascribed to proteins or genes. As a result, the past decade has seen metabolomics play an increasingly important role in unifying the different omics views of cancer. In particular, metabolomics has helped to characterize the cancer exposome, to reveal the cancer metabolome, and to identify new members of the cancer genome. In addition to characterizing these cancer “omes”, metabolomics has also helped us understand more about their molecular mechanisms. For instance, metabolomics has shown how different members of the cancer exposome function and how these environmental molecules, lifestyles, or microbial exposures lead to genetic instability, ROS production, or tumor promoting inflammation. Metabolomics has also shown that many of these external mutagens can be metabolized to even more potent carcinogens within the body and that they have cancer-inducing effects that extend far beyond simple mutagenesis. Similarly, metabolomics has helped identify the many endogenously produced chemicals that amplify the effects of exogenous exposures (i.e., oncometabolites). The carcinogenic mechanisms behind most oncometabolites have also been revealed through metabolomics (and other omics fields) and these studies have shown that a large number of genes and proteins play a role in their production. The identification of these oncometabolite genes (oncogenes and tumor suppressors) has helped further refine and define the cancer genome. It has also helped link the cancer metabolome to the cancer genome.

**Figure 2:**
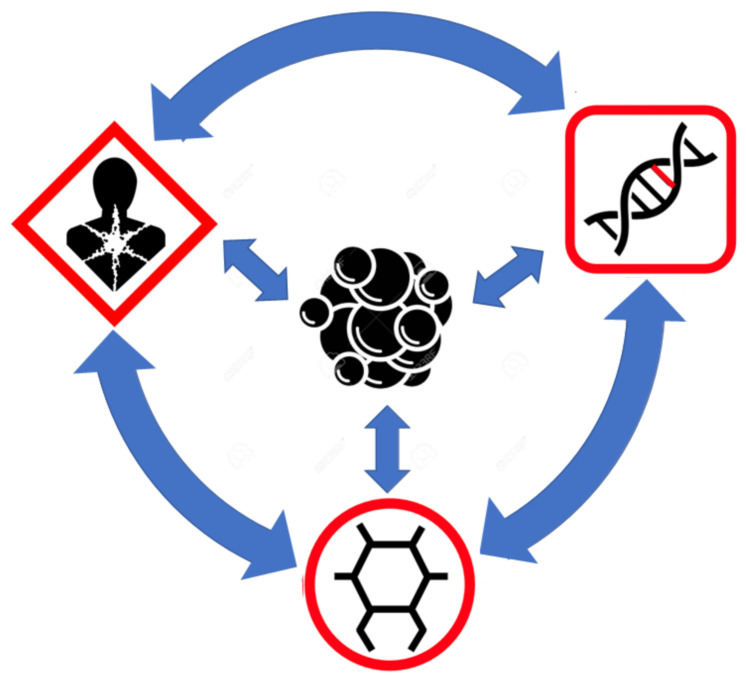
An interconnected, multi-omics view of cancer. In this view the exposome, the genome, and the metabolome all contribute individually to the development of cancer (arrows pointing inward). Any of these three “omes” is capable of initiating oncogenic transformation. Once transformed, the growing tumor also modifies the surrounding metabolome, exposome, and genome through its own altered metabolism and its own altered tumor microenvironment (arrows pointing outward). This constant feedback seems to amplify many of the genetic/metabolic drivers of cancer and helps manifest most of the hallmarks of cancer. The arrows connecting the genome with the exposome, and the metabolome are intended to show that each of these “omes” impacts the other. The genome can affect the exposome (or one’s proclivity to certain lifestyles or exposures), the exposome can impact the genome (through mutagenesis or ROS induced modifications). Likewise, the metabolome can alter the exposome (via chemical or enzymatic processes), while the exposome can also impact the metabolome (via catabolic or anabolic processes on exposure agents). Finally, the metabolome can affect the genome through epigenetic and direct genetic modifications, while the genome can alter the metabolome through genetically driven metabolic reprogramming.

The fact that endogenous metabolites, on their own, have been shown to induce cancer via direct mutagenesis or the induction of genetic instability argues against the sequential process of carcinogenesis depicted in Figure 1. This particular figure suggests the exposome (alone) alters the genome which, then, alters the cells and their metabolome, which, then, leads to cancer. However, as shown in this review, the exposome, the metabolome, and even the genome can lead to genetic alterations that initiate oncogenesis. Furthermore, there is both crosstalk between the different “omes” and feedback between the different “omes” which can help amplify and sustain oncogenic signals. Therefore, a more integrated, less linear view of carcinogenesis must be considered. This revised, “big picture” view is depicted in Figure 2.

As shown in Figure 2, the exposome, the genome, and the metabolome can all contribute to the development of cancer by initiating oncogenic transformation. Once transformed, cancer cells continue to modify their internal metabolomes and genomes as well as the surrounding exposome through their own altered (genetically encoded) metabolism. This feedback amplifies many of the initial genetic/metabolic drivers and helps manifest most of the hallmarks of cancer. In addition to the direct influence on cancer cells, the genome, the exposome, and the metabolome can also affect each other. This is explained in more detail in the figure legend. It is likely that this multi-omics crosstalk can either increase or reduce one’s risk for developing cancer.

While this big picture, multi-omics view of cancer may seem somewhat more complex than the usual cause and effect models associated with most cancer theories, it actually helps to unify many of the historically disparate views on cancer. It also explains why a single type of cancer treatment (for example, one that only targets the genome) or why a single type of prevention strategy (for example, one that only targets the exposome) has generally been unsuccessful arresting or preventing most cancers. Rather, multi-pronged therapeutic approaches and multi-pronged prevention strategies must be used–not unlike those that have been so successfully used to treat and prevent COVID-19 or to combat AIDS.

As mentioned at the beginning of this review, cancer is a disease of many disguises. These disguises have confounded and confused physicians and scientists for centuries. Thankfully, through the increased use of metabolomics and the integration of multiple omics techniques in cancer research, great strides have been made in learning to distinguish cancer’s many different masks and manifestations. By recognizing cancer as a multi-faced, multifaceted disorder and learning to combat each of its different manifestations with more multi-pronged prevention strategies and more multi-pronged therapies, we should be hopeful that someday we will tame this cunning and deceptive disease.

## Figures and Tables

**Table 1:** The genetic contributions to cancer. Prevalence of heritable cancers as compared with the estimated heritability of cancers.

Cancer Type	Number of Cases/Year in USA (2021)	Germline Prevalence (%)	Familial Prevalence (%)	GWAS Heritability (%)	Twin Heritability (%)
Breast	281,550 [55]	5.7–11.1 [21,22,23] Ave. = 8.4	13.6 [20]	9.7 [40]	31 [18]
Prostate	248,530 [55]	2.9–17.2 [24,25,26] Ave. = 10.1	20.2 [20]	11.0 [41]	57 [18]
Lung	235,760 [55]	0.3–1.4 [27,28,29] Ave. = 0.9	8.7 [20]	0.7 [42]	18 [18]
Colorectal	149,500 [55]	3.5–7.5 [30,31] Ave. = 5.5	12.8 [20]	1.2 [43]	15 [18]
Melanoma	106,110 [55]	1.9–3.1 [32,33] Ave. = 2.5	4.9 [20]	0.9 [44]	58 [18]
Bladder	83,730 [55]	8.9 [34]	5.4 [20]	0.9 [45]	30 [18]
Non-Hodgkin Lymphoma	81,560 [55]	7.7 [35]	2.9 [20]	0.7 [46]	25 [19]
Kidney (RCC)	76,080 [55]	7.9 [36]	3.6 [20]	0.6 [47]	38 [18]
Endometrial Uterine	66,570 [55]	4.6 [37]	4.1 [20]	0.6 [48]	27 [18]
Pancreatic	60,430 [55]	3.9 [38]	3.7 [20]	0.6 [49]	36 [52]
Thyroid	44,280 [55]	NA	3.5 [20]	1.5 [50]	53 [53]
Liver/bile Duct	42,230 [55]	5.9 [39]	2.6 [20]	1.7 [51]	30 [54]
Range		0.3–17.2	2.6–20.2	0.6–11.0	15.0–57.0
Case-weighted average		6.2	10.2	4.3	34.2

**Table 2:** Risk factors and causes of cancer deaths in the United States.

Cause	Percentage of Cancer Deaths in the US (%)	References
Age (>65)	72.0	[86]
Smoking	28.8–31.7	[87,88,89]
Obesity	7.0	[90]
Germline mutations/heritable cancers	3.3–5.9	This paper
Infectious agents	5.9	[91]
Alcohol	3.5–4.0	[90,92]
Radon exposure	3.5	[93]
Outdoor air pollution (PM 2.5)	3.1	[94,95]
Adverse effects of cancer treatment	2.8	[96]
Low fruit/vegetable diet	2.7	[85]
Diabetes	2.5	[97]
Physical inactivity	2.2	[85]
UV exposure	1.5	[85,98]
Red meat consumption	0.5–1.4	[99]
Diesel fumes	1.3	[100]
Second-hand smoke	1.2	[101]
Low fiber intake	0.9	[85]
Processed meat intake	0.7–0.8	[85,99]
Asbestos exposure	0.7	[102,103]
Low calcium and iodine intake	0.5	[85,104]
Miscellaneous occupational chemical exposures	0.5	[105]
Ionizing radiation (CT scans, radiotherapy)	0.3	[106]
**Total (excluding age)**	**73.4–80.4**	

**Table 3:** Oncometabolites (traditional and non-traditional), their associations with different cancers, their mechanisms, and cancer hallmark associations.

Oncometabolite	Cancer(s)	Mechanisms	Cancer HallMarks	Reference
Arginine	Ovarian cancer, pancreatic cancer, glioma, acute lymphoblastic leukemia (ALL), lung cancer, bladder cancer, colon cancer	Metastasis signaling, cell growth signaling (mTOR), reduced autophagy, DNA instability, mitochondrial dysfunction, Angiogenesis, anti-apoptosis, immune suppression	Evading growth suppressors, sustained proliferative signaling, genome instability, resisting cell death, replicative immortality, evading immune destruction, inducing angiogenesis	[135]
Asparagine	Acute lymphoblastic leukemia, breast cancer, lung cancer	Anti-apoptosis, Cell growth signaling, metastasis signaling	Dysregulated metabolism, resisting cell death, sustained proliferative signaling, evading growth suppressors, activating invasion and metastasis	[136]
Choline	Prostate cancer, brain cancer, breast cancer	Hypoxic, hyperglycemic growth, epigenetic modifications	Dysregulated metabolism, genome instability, sustained proliferative signaling	[137]
Cystathionine	Breast cancer	ROS protection, anti-apoptosis	replicative immortality, resisting cell death	[138]
Deoxycholic acid	Colon cancer	Mitochondrial dysfunction, ROS production, anti-apoptosis, proinflammation	evading growth suppressors, tumor promoting inflammation, resisting cell death	[139]
Diacetylspermine	Neuroblastoma, liver cancer, breast cancer, colon cancer, lung cancer	Anti-apoptosis, cell growth signaling, immune suppression	Resisting cell death, sustained proliferative signaling, evading immune destruction	[140]
Estradiol	Ovarian cancer, endometrial cancer breast cancer	Cell growth signaling, metastasis signaling	Sustained proliferative signaling, activating invasion and metastasis	[141]
Fumarate	Praganglioma, pheochromocytoma, renal cell carcinoma	Epigenetic modifications, protein modification	Dysregulated metabolism, genome instability, sustained proliferative signaling	[142]
N-acetyl-D-glucosamine	Systemic mastocytosis	Cell growth signaling, proinflammation	Sustained proliferative signaling, tumor promoting inflammation	[143]
Glucose	Most cancers	Hyperglycemic growth, aerobic glycolysis, protein modification	Dysregulated metabolism, sustained proliferative signaling, replicative immortality	[144]
Glutamine	Glioma, acute myeloid leukemia, lung cancer, breast cancer	Glutaminolysis, ROS protection, cell growth signaling (mTOR), reduced autophagy, DNA instability, mitochondrial dysfunction, metastasis signaling	Dysregulated metabolism, replicative immortality, sustained proliferative signaling, evading growth suppressors, genome instability, resisting cell death, activating invasion and metastasis	[145]
D-2-hydroxy-glutarate	Glioma, acute myeloid leukemia, prostate cancer, colon cancer	Epigenetic modifications, hypoxic, hyperglycemic growth, cell growth signaling (mTOR), ROS production, angiogenesis, immune suppression	Dysregulated metabolism, genome instability, inducing angiogenesis, resisting cell death, sustained proliferative signaling, evading immune destruction	[10]
L-2-hydroxy-glutarate	Renal cell carcinoma	Epigenetic modifications, hypoxic, hyperglycemic growth, cell growth signaling (mTOR), immune suppression, ROS production	Dysregulated metabolism, genome instability, resisting cell death, sustained proliferative signaling, evading immune destruction	[146]
Glycine	Lung cancer, glioma	hyperglycemic growth, aerobic glycolysis, epigenetic modifications	Dysregulated metabolism, genome instability	[147]
Homocysteine	Most cancers	Reduced DNA repair, proinflammation, epigenetic modifications	Genome instability, tumor promoting inflammation	[148]
Hypotaurine	Glioma	Epigenetic modifications, hypoxic, hyperglycemic growth	Dysregulated metabolism, genome instability, sustained proliferative signaling	[149]
Isoleucine	Lung cancer, glioma, breast cancer, glioma, endometrial cancer	Cell growth signaling (mTOR), reduced autophagy, DNA instability, mitochondrial dysfunction	Evading growth suppressors, sustained proliferative signaling, genome instability, resisting cell death, replicative immortality	[150]
Kynurenine	Colon cancer, lung cancer, prostate cancer, glioma, breast cancer	Cell growth signaling, immune suppression, metastasis signaling, proinflammation	Sustained proliferative signaling, evading immune destruction, tumor promoting inflammation, activating invasion and metastasis	[151]
Lactate	Most cancers	Metastasis signaling, immune suppression, angiogenesis, anti-apoptosis, proinflammation	Dysregulated metabolism, activating invasion and metastasis, inducing angiogenesis, evading immune destruction, tumor promoting inflammation	[152]
Leucine	Lung cancer, glioma, breast cancer, glioma, endometrial cancer	Cell growth signaling (mTOR), reduced autophagy, DNA instability, mitochondrial dysfunction	Evading growth suppressors, sustained proliferative signaling, genome instability, resisting cell death, replicative immortality	[150]
Lithocholic acid	Colon cancer	Mitochondrial dysfunction, ROS production, anti-apoptosis, proinflammation	Evading growth suppressors, tumor promoting inflammation, resisting cell death	[139]
Methionine	Colon cancer, pancreatic cancer, glioma, endometrial cancer	Cell growth signaling (mTOR), reduced autophagy, epigenetic modifications, mitochondrial dysfunction, anti-apoptosis, Immune suppression	Evading growth suppressors, sustained proliferative signaling, genome instability, resisting cell death, replicative immortality, evading immune destruction	[153]
Methylglyoxal	Breast cancer	Metastasis signaling, protein modification, proinflammation	Dysregulated metabolism, activating invasion and metastasis, tumor promoting inflammation	[154]
Methylmalonate	Liver cancer	Mitochondrial dysfunction, ROS production, DNA instability, proinflammation	Dysregulated metabolism, resisting cell death, genome instability, tumor promoting inflammation	[155]
Nitric Oxide	Lung cancer, colon cancer, breast cancer, pancreatic cancer, prostate Cancer	Angiogenesis, metastasis signaling, DNA instability, proinflammation	Inducing angiogenesis, activating invasion and metastasis, genome instability, tumor promoting inflammation	[156]
Progesterone	Ovarian cancer	Cell growth signaling, metastasis signaling	Sustained proliferative signaling, activating invasion and metastasis	[141]
Putrescine	Neuroblastoma, liver cancer, breast cancer, colon cancer, lung cancer	Anti-apoptosis, cell growth signaling, immune suppression	Resisting cell death, sustained proliferative signaling, evading immune destruction	[140]
4-Pyridone-3-carboxamide-1-beta-D-ribonucleoside	Lung cancer, breast cancer	Metastasis signaling	Activating invasion and metastasis	[157]
SAICAR	Oral cancer, most cancers	Aerobic glycolysis, PKM2 signaling, cell growth signaling	Dysregulated metabolism, sustained proliferative signaling	[158]
Sarcosine	Prostate cancer	Epigenetic modifications, metastasis signaling	Dysregulated metabolism, genome instability, activating invasion and metastasis	[159]
Serine	Breast cancer, glioma, cervical cancer	Hyperglycemic growth, aerobic glycolysis, PKM2 signaling	dysregulated metabolism, replicative immortality	[160]
Spermidine	Neuroblastoma, liver cancer, breast cancer, colon cancer, lung cancer	Anti-apoptosis, cell growth signaling, immune suppression	resisting cell death, sustained proliferative signaling, evading immune destruction	[140]
Spermine	Neuroblastoma, liver cancer, breast cancer, colon cancer, lung cancer	Anti-apoptosis, cell growth signaling, immune suppression	Resisting cell death, sustained proliferative signaling, evading immune destruction	[140]
Succinate	Praganglioma, pheochromocytoma, renal cell carcinoma	Epigenetic modifications, hypoxic, hyperglycemic growth, angiogenesis, proinflammation, cell growth signaling	Dysregulated metabolism, genome instability, tumor promoting inflammation, inducing angiogenesis, sustained proliferative signaling	[161]
Succinyl-acetoacetate	Liver cancer	Protein modification, cell growth signaling	Dysregulated metabolism, Genome instability, sustained proliferative signaling	[162]
Succinyl-acetone	Liver cancer	Protein modification, cell growth signaling	Dysregulated metabolism, genome instability, sustained proliferative signaling	[162]
Uric acid	Liver cancer, lung cancer, liver cancer, bladder cancer, prostate cancer	Proinflammation, ROS protection	Tumor promoting inflammation, replicative immortality	[163]
Valine	Lung cancer, glioma, breast cancer, glioma, endometrial cancer	Cell growth signaling (mTOR), reduced autophagy, DNA instability, mitochondrial dysfunction	Evading growth suppressors, sustained proliferative signaling, genome instability, resisting cell death, replicative immortality	[150]

## References

[1] Brown G. (2021). Oncogenes, Proto-oncogenes, and lineage restriction of cancer stem cells. Int. J. Mol. Sci..

[2] Mukherjee S. (2011). The Emperor of All Maladies.

[3] Hadju S.I. (2006). Thoughts about the cause of cancer. Cancer.

[4] Faguet G.B. (2015). A brief history of cancer: Age-old milestones underlying our current knowledge database. Int. J. Cancer.

[5] Carrel A., Ebeling A.H. (1926). The transformation of monocytes into fibroblasts through the action of Rous virus. J. Exp. Med..

[6] Warburg O., Wind F., Negelein E. (1927). The metabolism of tumors in the body. J. Gen. Physiol..

[7] Busk T., Clemmesen J., Nielsen A. (1948). Twin studies and other genetical investigations in the Danish Cancer Registry. Br. J. Cancer.

[8] Comings D.E. (1973). A general theory of carcinogenesis. Proc. Natl. Acad. Sci. USA.

[9] Fiala S. (1968). The cancer cell as a stem cell unable to differentiate. A theory of carcinogenesis. Neoplasma.

[10] Dang L., White D.W., Gross S., Bennett B.D., Bittinger M.A., Driggers E.M., Fantin V.R., Jang H.G., Jin S., Keenan M.C. (2009). Cancer-associated IDH1 mutations produce 2-hydroxyglutarate. Nature.

[11] Hanahan D., Weinberg R.A. (2011). Hallmarks of cancer: The next generation. Cell.

[12] Soto A.M., Sonnenschein C. (2011). The tissue organization field theory of cancer: A testable replacement for the somatic mutation theory. Bioessays.

[13] Seyfried T.N., Chinopoulos C. (2021). Can the mitochondrial metabolic theory explain better the origin and management of cancer than can the somatic mutation theory?. Metabolites.

[14] Wishart D.S. (2015). Is cancer a genetic disease or a metabolic disease?. EBioMedicine.

[15] Durham H.W. (1954). Familial cancer of the colon. West. J. Surg. Obstet. Gynecol..

[16] Nagy R., Sweet K., Eng C. (2004). Highly penetrant hereditary cancer syndromes. Oncogene.

[17] Easton D., Peto J. (1990). The contribution of inherited predisposition to cancer incidence. Cancer Surv..

[18] Mucci L.A., Hjelmborg J.B., Harris J.R., Czene K., Havelick D.J., Scheike T., Graff R.E., Holst K., Möller S., Unger R.H. (2016). Nordic twin study of cancer (NorTwinCan) collaboration. Familial risk and heritability of cancer among twins in nordic countries. JAMA.

[19] Clemmensen S.B., Harris J.R., Mengel-From J., Bonat W.H., Frederiksen H., Kaprio J., Hjelmborg J.V.B. (2021). Familial risk and heritability of hematologic malignancies in the nordic twin study of cancer. Cancers.

[20] Hemminki K., Sundquist J., Bermejo J.L. (2008). How common is familial cancer?. Ann. Oncol..

[21] Chen B., Zhang G., Li X., Ren C., Wang Y., Li K., Mok H., Cao L., Wen L., Jia M. (2020). Comparison of BRCA versus non-BRCA germline mutations and associated somatic mutation profiles in patients with unselected breast cancer. Aging.

[22] Momozawa Y., Iwasaki Y., Parsons M.T., Kamatani Y., Takahashi A., Tamura C., Katagiri T., Yoshida T., Nakamura S., Sugano K. (2018). Germline pathogenic variants of 11 breast cancer genes in 7051 Japanese patients and 11,241 controls. Nat. Commun..

[23] Sun J., Meng H., Yao L., Lv M., Bai J., Zhang J., Wang L., Ouyang T., Li J., Wang T. (2017). Germline mutations in cancer susceptibility genes in a large series of unselected breast cancer patients. Clin. Cancer Res..

[24] Momozawa Y., Iwasaki Y., Hirata M., Liu X., Kamatani Y., Takahashi A., Sugano K., Yoshida T., Murakami Y., Matsuda K. (2020). Germline pathogenic variants in 7636 Japanese patients with prostate cancer and 12,366 controls. J. Natl. Cancer Inst..

[25] Pritchard C.C., Mateo J., Walsh M.F., De Sarkar N., Abida W., Beltran H., Garofalo A., Gulati R., Carreira S., Eeles R. (2016). Inherited DNA-repair gene mutations in men with metastatic prostate cancer. N. Engl. J. Med..

[26] Nicolosi P., Ledet E., Yang S., Michalski S., Freschi B., O’Leary E., Esplin E.D., Nussbaum R.L., Sartor O. (2019). Prevalence of germline variants in prostate cancer and implications for current genetic testing guidelines. JAMA Oncol..

[27] Hu X., Yang D., Li Y., Li L., Wang Y., Chen P., Xu S., Pu X., Zhu W., Deng P. (2019). Prevalence and clinical significance of pathogenic germline BRCA1/2 mutations in Chinese non-small cell lung cancer patients. Cancer Biol. Med..

[28] Liu M., Liu X., Suo P., Gong Y., Qu B., Peng X., Xiao W., Li Y., Chen Y., Zeng Z. (2020). The contribution of hereditary cancer-related germline mutations to lung cancer susceptibility. Transl. Lung Cancer Res..

[29] Yang J., Li H., Lim B., Li W., Guo Q., Hu L., Song Z., Zhou B. (2021). Profiling oncogenic germline mutations in unselected Chinese lung cancer patients. Front. Oncol..

[30] Fujita M., Liu X., Iwasaki Y., Terao C., Mizukami K., Kawakami E., Takata S., Inai C., Aoi T., Mizukoshi M. (2020). Population-based Screening for hereditary colorectal cancer variants in Japan. Clin. Gastroenterol. Hepatol..

[31] DeRycke M.S., Gunawardena S., Balcom J.R., Pickart A.M., Waltman L.A., French A.J., McDonnell S., Riska S.M., Fogarty Z.C., Larson M.C. (2017). Targeted sequencing of 36 known or putative colorectal cancer susceptibility genes. Mol. Genet. Genomic Med..

[32] Aoude L.G., Gartside M., Johansson P., Palmer J.M., Symmons J., Martin N.G., Montgomery G.W., Hayward N.K. (2015). Prevalence of germline BAP1, CDKN2A and CDK4 mutations in an Australian population-based sample of cutaneous melanoma cases. Twin Res. Hum. Genet..

[33] Casula M., Colombino M., Satta M.P., Cossu A., Lissia A., Budroni M., Simeone E., Calemma R., Loddo C., Caracò C. (2007). Factors predicting the occurrence of germline mutations in candidate genes among patients with cutaneous malignant melanoma from South Italy. Eur. J. Cancer..

[34] Nassar A.H., Abou Alaiwi S., AlDubayan S.H., Moore N., Mouw K.W., Kwiatkowski D.J., Choueiri T.K., Curran C., Berchuck J.E., Harshman L.C. (2020). Prevalence of pathogenic germline cancer risk variants in high-risk urothelial carcinoma. Genet. Med..

[35] Scott A.J., Tokaz M.C., Jacobs M.F., Chinnaiyan A.M., Phillips T.J., Wilcox R.A. (2021). Germline variants discovered in lymphoma patients undergoing tumor profiling: A case series. Fam. Cancer.

[36] Abou Alaiwi S., Nassar A.H., Adib E., Groha S.M., Akl E.W., McGregor B.A., Esplin E.D., Yang S., Hatchell K., Fusaro V. (2021). Trans-ethnic variation in germline variants of patients with renal cell carcinoma. Cell Rep..

[37] Long B., Lilyquist J., Weaver A., Hu C., Gnanaolivu R., Lee K.Y., Hart S.N., Polley E.C., Bakkum-Gamez J.N., Couch F.J. (2019). Cancer susceptibility gene mutations in type I and II endometrial cancer. Gynecol. Oncol..

[38] Shindo K., Yu J., Suenaga M., Fesharakizadeh S., Cho C., Macgregor-Das A., Siddiqui A., Witmer P.D., Tamura K., Song T.J. (2017). Deleterious germline mutations in patients with apparently sporadic pancreatic adenocarcinoma. J. Clin. Oncol..

[39] Mezina A., Philips N., Bogus Z., Erez N., Xiao R., Fan R., Olthoff K.M., Reddy K.R., Samadder N.J., Nielsen S.M. (2021). Multigene panel testing in individuals with hepatocellular carcinoma identifies pathogenic germline variants. JCO Precis. Oncol..

[40] Michailidou K., Beesley J., Lindstrom S., Canisius S., Dennis J., Lush M.J., Maranian M.J., Bolla M.K., Wang Q., Shah M. (2015). Genome-wide association analysis of more than 120,000 individuals identifies 15 new susceptibility loci for breast cancer. Nat. Genet..

[41] Teerlink C.C., Leongamornlert D., Dadaev T., Thomas A., Farnham J., Stephenson R.A., Riska S., McDonnell S.K., Schaid D.J., Catalona W.J. (2016). Genome-wide association of familial prostate cancer cases identifies evidence for a rare segregating haplotype at 8q24.21. Hum. Genet..

[42] Hu Z., Wu C., Shi Y., Guo H., Zhao X., Yin Z., Yang L., Dai J., Hu L., Tan W. (2011). A genome-wide association study identifies two new lung cancer susceptibility loci at 13q12.12 and 22q12.2 in Han Chinese. Nat. Genet..

[43] Zhang B., Jia W.H., Matsuda K., Kweon S.S., Matsuo K., Xiang Y.B., Shin A., Jee S.H., Kim D.H., Cai Q. (2014). Large-scale genetic study in East Asians identifies six new loci associated with colorectal cancer risk. Nat. Genet..

[44] Bishop D.T., Demenais F., Iles M.M., Harland M., Taylor J.C., Corda E., Randerson-Moor J., Aitken J.F., Avril M.F., Azizi E. (2009). Genome-wide association study identifies three loci associated with melanoma risk. Nat. Genet..

[45] Figueroa J.D., Ye Y., Siddiq A., Garcia-Closas M., Chatterjee N., Prokunina-Olsson L., Cortessis V.K., Kooperberg C., Cussenot O., Benhamou S. (2014). Genome-wide association study identifies multiple loci associated with bladder cancer risk. Hum. Mol. Genet..

[46] Tan D.E., Foo J.N., Bei J.X., Chang J., Peng R., Zheng X., Wei L., Huang Y., Lim W.Y., Li J. (2013). Genome-wide association study of B cell non-Hodgkin lymphoma identifies 3q27 as a susceptibility locus in the Chinese population. Nat. Genet..

[47] Purdue M.P., Johansson M., Zelenika D., Toro J.R., Scelo G., Moore L.E., Prokhortchouk E., Wu X., Kiemeney L.A., Gaborieau V. (2011). Genome-wide association study of renal cell carcinoma identifies two susceptibility loci on 2p21 and 11q13.3. Nat. Genet..

[48] Cheng T.H., Thompson D.J., O’Mara T.A., Painter J.N., Glubb D.M., Flach S., Lewis A., French J.D., Freeman-Mills L., Church D. (2016). Five endometrial cancer risk loci identified through genome-wide association analysis. Nat. Genet..

[49] Childs E.J., Mocci E., Campa D., Bracci P.M., Gallinger S., Goggins M., Li D., Neale R.E., Olson S.H., Scelo G. (2015). Common variation at 2p13.3, 3q29, 7p13 and 17q25.1 associated with susceptibility to pancreatic cancer. Nat. Genet..

[50] Gudmundsson J., Sulem P., Gudbjartsson D.F., Jonasson J.G., Masson G., He H., Jonasdottir A., Sigurdsson A., Stacey S.N., Johannsdottir H. (2012). Discovery of common variants associated with low TSH levels and thyroid cancer risk. Nat. Genet..

[51] Li S., Qian J., Yang Y., Zhao W., Dai J., Bei J.X., Foo J.N., McLaren P.J., Li Z., Yang J. (2012). GWAS identifies novel susceptibility loci on 6p21.32 and 21q21.3 for hepatocellular carcinoma in chronic hepatitis B virus carriers. PLoS Genet..

[52] Chen F., Childs E.J., Mocci E., Bracci P., Gallinger S., Li D., Neale R.E., Olson S.H., Scelo G., Bamlet W.R. (2019). Analysis of heritability and genetic architecture of pancreatic cancer: A PanC4 study. Cancer Epidemiol. Biomark. Prev..

[53] Czene K., Lichtenstein P., Hemminki K. (2002). Environmental and heritable causes of cancer among 9.6 million individuals in the Swedish family-cancer satabase. Int. J. Cancer.

[54] Turati F., Edefonti V., Talamini R., Ferraroni M., Malvezzi M., Bravi F., Franceschi S., Montella M., Polesel J., Zucchetto A. (2012). Family history of liver cancer and hepatocellular carcinoma. Hepatology.

[55] Seigel R.L., Miller K.D., Fuchs H.E., Jemal A. (2021). Cancer statistics. CA Cancer J. Clin..

[56] Anand P., Kunnumakkara A.B., Sundaram C., Harikumar K.B., Tharakan S.T., Lai O.S., Sung B., Aggarwal B.B. (2008). Cancer is a preventable disease that requires major lifestyle changes. Pharm. Res..

[57] Freeman H.J. (2008). Colorectal cancer risk in Crohn’s disease. World J. Gastroenterol..

[58] Giovannucci E., Harlan D.M., Archer M.C., Bergenstal R.M., Gapstur S.M., Habel L.A., Pollak M., Regensteiner J.G., Yee D. (2010). Diabetes and cancer: A consensus report. Diabetes Care.

[59] Frank C., Sundquist J., Yu H., Hemminki A., Hemminki K. (2017). Concordant and discordant familial cancer: Familial risks, proportions and population impact. Int. J. Cancer.

[60] Beck T., Shorter T., Brookes A.J. (2020). GWAS Central: A comprehensive resource for the discovery and comparison of genotype and phenotype data from genome-wide association studies. Nucleic Acids Res..

[61] Patron J., Serra-Cayuela A., Han B., Li C., Wishart D.S. (2019). Assessing the performance of genome-wide association studies for predicting disease risk. PLoS ONE.

[62] Segalowitz S.J. (1999). Why twin studies really don’t tell us much about human heritability. Behav. Brain Sci..

[63] Young A.I. (2019). Solving the missing heritability problem. PLoS Genet..

[64] Krishna Kumar S., Feldman M.W., Rehkopf D.H., Tuljapurkar S. (2016). Limitations of GCTA as a solution to the missing heritability problem. Proc. Natl. Acad. Sci. USA.

[65] Hallmayer J., Cleveland S., Torres A., Phillips J., Cohen B., Torigoe T., Miller J., Fedele A., Collins J., Smith K. (2011). Genetic heritability and shared environmental factors among twin pairs with autism. Arch. Gen. Psychiatry.

[66] Sandin S., Lichtenstein P., Kuja-Halkola R., Hultman C., Larsson H., Reichenberg A. (2017). The heritability of autism spectrum disorder. JAMA.

[67] De Groot P.M., Wu C.C., Carter B.W., Munden R.F. (2018). The epidemiology of lung cancer. Transl. Lung Cancer Res..

[68] Sun X., Zhang N., Yin C., Zhu B., Li X. (2020). Ultraviolet radiation and melanomagenesis: From mechanism to immunotherapy. Front. Oncol..

[69] Vogelstein B., Papadopoulos N., Velculescu V.E., Zhou S., Diaz L.A., Kinzler K.W. (2013). Cancer genome landscapes. Science.

[70] Bailey M.H., Tokheim C., Porta-Pardo E., Sengupta S., Bertrand D., Weerasinghe A., Colaprico A., Wendl M.C., Kim J., Reardon B. (2018). Comprehensive characterization of cancer driver genes and mutations. Cell.

[71] Tate J.G., Bamford S., Jubb H.C., Sondka Z., Beare D.M., Bindal N., Boutselakis H., Cole C.G., Creatore C., Dawson E. (2019). COSMIC: The catalogue of somatic mutations in cancer. Nucleic Acids Res..

[72] Knudson A.G. (1971). Mutation and cancer: Statistical study of retinoblastoma. Proc. Natl. Acad. Sci. USA.

[73] Wild C.P. (2005). Complementing the genome with an "exposome": The outstanding challenge of environmental exposure measurement in molecular epidemiology. Cancer Epidemiol. Biomark. Prev..

[74] Shah D.J., Sachs R.K., Wilson D.J. (2012). Radiation-induced cancer: A modern view. Br. J. Radiol..

[75] Campbell T.C. (2017). The past, present, and future of nutrition and cancer: Part 1-was a nutritional association acknowledged a century ago?. Nutr. Cancer.

[76] Neveu V., Moussy A., Rouaix H., Wedekind R., Pon A., Knox C., Wishart D.S., Scalbert A. (2017). Exposome-explorer: A manually-curated database on biomarkers of exposure to dietary and environmental factors. Nucleic Acids Res..

[77] Neveu V., Nicolas G., Salek R.M., Wishart D.S., Scalbert A. (2020). Exposome-explorer 2.0: An update incorporating candidate dietary biomarkers and dietary associations with cancer risk. Nucleic Acids Res..

[78] Liao J.B. (2006). Viruses and human cancer. Yale J. Biol. Med..

[79] Mandong B.M., Ngbea J.A., Raymond V. (2013). Role of parasites in cancer. Niger. J. Med..

[80] Parsonnet J. (1995). Bacterial infection as a cause of cancer. Environ. Health Perspect..

[81] Maciejewska A., Wojtczak J., Bielichowska-Cybula G., Domańska A., Dutkiewicz J., Mołocznik A. (1993). Biological effect of wood dust. Med. Pracy.

[82] Chen Y., Tong Y., Yang C., Gan Y., Sun H., Bi H., Cao S., Yin X., Lu Z. (2015). Consumption of hot beverages and foods and the risk of esophageal cancer: A meta-analysis of observational studies. BMC Cancer.

[83] Koritala B.S.C., Porter K.I., Arshad O.A., Gajula R.P., Mitchell H.D., Arman T., Manjanatha M.G., Teeguarden J., Van Dongen H.P.A., McDermott J.E. (2021). Night shift schedule causes circadian dysregulation of DNA repair genes and elevated DNA damage in humans. J. Pineal Res..

[84] Hayes J.D., Dinkova-Kostova A.T., Tew K.D. (2020). Oxidative stress in cancer. Cancer Cell..

[85] Islami F., Goding Sauer A., Miller K.D., Siegel R.L., Fedewa S.A., Jacobs E.J., McCullough M.L., Patel A.V., Ma J., Soerjomataram I. (2018). Proportion and number of cancer cases and deaths attributable to potentially modifiable risk factors in the United States. CA Cancer J. Clin..

[86] An Update on Cancer Deaths in the United States. https://www.cdc.gov/cancer/dcpc/research/update-on-cancer-deaths/index.htm.

[87] Siegel R.L., Jacobs E.J., Newton C.C., Feskanich D., Freedman N.D., Prentice R.L., Jemal A. (2015). Deaths due to cigarette smoking for 12 smoking-related cancers in the United States. JAMA Intern. Med..

[88] Shalo Wilmont S. (2015). Cigarettes still cause a third of U.S. cancer deaths. Am. J. Nurs..

[89] Jacobs E.J., Newton C.C., Carter B.D., Feskanich D., Freedman N.D., Prentice R.L., Flanders W.D. (2015). What proportion of cancer deaths in the contemporary United States is attributable to cigarette smoking?. Ann. Epidemiol..

[90] Alvarnas A., Alvarnas J. (2019). Obesity and cancer risk: A public health crisis. Am. J. Manag. Care.

[91] De Martel C., Georges D., Bray F., Ferlay J., Clifford G.M. (2020). Global burden of cancer attributable to infections in 2018: A worldwide incidence analysis. Lancet Glob. Health..

[92] Nelson D.E., Jarman D.W., Rehm J., Greenfield T.K., Rey G., Kerr W.C., Miller P., Shield K.D., Ye Y., Naimi T.S. (2013). Alcohol-attributable cancer deaths and years of potential life lost in the United States. Am. J. Public Health.

[93] Cao X., MacNaughton P., Laurent J.C., Allen J.G. (2017). Radon-induced lung cancer deaths may be overestimated due to failure to account for confounding by exposure to diesel engine exhaust in BEIR VI miner studies. PLoS ONE.

[94] Turner M.C., Andersen Z.J., Baccarelli A., Diver W.R., Gapstur S.M., Pope C.A., Prada D., Samet J., Thurston G., Cohen A. (2020). Outdoor air pollution and cancer: An overview of the current evidence and public health recommendations. CA Cancer J. Clin..

[95] Grant W.B. (2009). Air pollution in relation to U.S. cancer mortality rates: An ecological study; likely role of carbonaceous aerosols and polycyclic aromatic hydrocarbons. Anticancer Res..

[96] Sunshine J.E., Meo N., Kassebaum N.J., Collison M.L., Mokdad A.H., Naghavi M. (2019). Association of adverse effects of medical treatment with mortality in the United States: A secondary analysis of the global burden of diseases, injuries, and risk factors study. JAMA Netw. Open.

[97] Harding J.L., Andes L.J., Gregg E.W., Cheng Y.J., Weir H.K., Bullard K.M., Burrows N.R., Imperatore G. (2020). Trends in cancer mortality among people with vs without diabetes in the USA, 1988–2015. Diabetologia.

[98] Guy G.P., Thomas C.C., Thompson T., Watson M., Massetti G.M., Richardson L.C. (2015). Centers for disease control and prevention (CDC). Vital signs: Melanoma incidence and mortality trends and projections—United States, 1982–2030. MMWR Morb. Mortal. Wkly. Rep..

[99] Sinha R., Cross A.J., Graubard B.I., Leitzmann M.F., Schatzkin A. (2009). Meat intake and mortality: A prospective study of over half a million people. Arch. Intern. Med..

[100] Vermeulen R., Silverman D.T., Garshick E., Vlaanderen J., Portengen L., Steenland K. (2014). Exposure-response estimates for diesel engine exhaust and lung cancer mortality based on data from three occupational cohorts. Environ. Health Perspect..

[101] Naeem Z. (2015). Second-hand smoke—Ignored implications. Int. J. Health Sci..

[102] Furuya S., Chimed-Ochir O., Takahashi K., David A., Takala J. (2018). Global asbestos disaster. Int. J. Environ. Res. Public Health.

[103] Nicholson W.J., Perkel G., Selikoff I.J. (1982). Occupational exposure to asbestos: Population at risk and projected mortality—1980–2030. Am. J. Ind. Med..

[104] Zimmermann M.B., Galetti V. (2015). Iodine intake as a risk factor for thyroid cancer: A comprehensive review of animal and human studies. Thyroid Res..

[105] Attributable Fraction: Example Cancers Due to Occupation in the US. http://www.occupationalcancer.ca/wp-content/uploads/2011/03/Steenland.pdf.

[106] Zallman L., Woolhandler S., Himmelstein D., Bor D.H., McCormick D. (2012). Computed tomography associated cancers and cancer deaths following visits to U.S. emergency departments. Int. J. Health Serv..

[107] Aunan J.R., Cho W.C., Søreide K. (2017). The biology of aging and cancer: A brief overview of shared and divergent molecular hallmarks. Aging Dis..

[108] Støer N.C., Botteri E., Thoresen G.H., Karlstad Ø., Weiderpass E., Friis S., Pottegård A., Andreassen B.K. (2021). Drug use and cancer risk: A drug-wide association study (DWAS) in Norway. Cancer Epidemiol. Biomark. Prev..

[109] Tu H., Wen C.P., Tsai S.P., Chow W.H., Wen C., Ye Y., Zhao H., Tsai M.K., Huang M., Dinney C.P. (2018). Cancer risk associated with chronic diseases and disease markers: Prospective cohort study. BMJ.

[110] Ong J.S., An J., Law M.H., Whiteman D.C., Neale R.E., Gharahkhani P., MacGregor S. (2018). Height and overall cancer risk and mortality: Evidence from a Mendelian randomisation study on 310,000 UK Biobank participants. Br. J. Cancer.

[111] Schwabe R.F., Jobin C. (2013). The microbiome and cancer. Nat. Rev. Cancer.

[112] Emmons K.M., Colditz G.A. (2017). Realizing the potential of cancer prevention—The role of implementation science. N. Engl. J. Med..

[113] Maeda H., Khatami M. (2018). Analyses of repeated failures in cancer therapy for solid tumors: Poor tumor-selective drug delivery, low therapeutic efficacy and unsustainable costs. Clin. Transl. Med..

[114] DeVita V.T., Chu E. (2008). A history of cancer chemotherapy. Cancer Res..

[115] Wishart D.S. (2016). Emerging applications of metabolomics in drug discovery and precision medicine. Nat. Rev. Drug Discov..

[116] Thompson C.B. (2009). Metabolic enzymes as oncogenes or tumor suppressors. N. Engl. J. Med..

[117] Pavlova N.N., Thompson C.B. (2016). The emerging hallmarks of cancer metabolism. Cell Metab..

[118] Dong Y., Tu R., Liu H., Qing G. (2020). Regulation of cancer cell metabolism: Oncogenic MYC in the driver’s seat. Signal Transduct. Target. Ther..

[119] Wishart D.S. (2019). Metabolomics for investigating physiological and pathophysiological processes. Physiol. Rev..

[120] Lunt S.Y., Vander Heiden M.G. (2011). Aerobic glycolysis: Meeting the metabolic requirements of cell proliferation. Ann. Rev. Cell Dev. Biol..

[121] Altenberg B., Greulich K.O. (2004). Genes of glycolysis are ubiquitously overexpressed in 24 cancer classes. Genomics.

[122] Shuch B., Linehan W.M., Srinivasan R. (2013). Aerobic glycolysis: A novel target in kidney cancer. Expert Rev. Anticancer Ther..

[123] Jin L., Alesi G.N., Kang S. (2016). Glutaminolysis as a target for cancer therapy. Oncogene..

[124] Goetzman E.S., Prochownik E.V. (2018). The role for Myc in coordinating glycolysis, oxidative phosphorylation, glutaminolysis, and fatty acid metabolism in normal and neoplastic tissues. Front. Endocrinol..

[125] Li A.M., Ye J. (2020). Reprogramming of serine, glycine and one-carbon metabolism in cancer. Biochim. Biophys. Acta Mol. Basis Dis..

[126] Porporato P.E. (2016). Understanding cachexia as a cancer metabolism syndrome. Oncogenesis.

[127] Argiles J., Costelli P., Carbo N., Lopezsoriano F. (1996). Branched-chain amino acid catabolism and cancer cachexia (review). Oncol. Rep..

[128] Aoyagi T., Terracina K.P., Raza A., Matsubara H., Takabe K. (2015). Cancer cachexia, mechanism and treatment. World J. Gastrointest. Oncol..

[129] Lieu E.L., Nguyen T., Rhyne S., Kim J. (2020). Amino acids in cancer. Exp. Mol. Med..

[130] Yang M., Soga T., Pollard P. (2013). J. Oncometabolites: Linking altered metabolism with cancer. J. Clin. Investig..

[131] Seok J., Yoon S.H., Lee S.H., Jung J.H., Lee Y.M. (2019). The oncometabolite d-2-hydroxyglutarate induces angiogenic activity through the vascular endothelial growth factor receptor 2 signaling pathway. Int. J. Oncol..

[132] Yang Z., Jiang B., Wang Y., Ni H., Zhang J., Xia J., Shi M., Hung L.M., Ruan J., Mak T.W. (2017). 2-HG inhibits necroptosis by stimulating DNMT1-dependent hypermethylation of the RIP3 promoter. Cell Rep..

[133] Richardson L.G., Choi B.D., Curry W.T. (2019). (R)-2-hydroxyglutarate drives immune quiescence in the tumor microenvironment of IDH-mutant gliomas. Transl. Cancer Res..

[134] Carbonneau M., Gagné L., Lalonde M.E., Germain M.A., Motorina A., Guiot M.C., Secco B., Vincent E.E., Tumber A., Hulea L. (2016). The oncometabolite 2-hydroxyglutarate activates the mTOR signalling pathway. Nat. Commun..

[135] Al-Koussa H., El Mais N., Maalouf H., Abi-Habib R., El-Sibai M. (2020). Arginine deprivation: A potential therapeutic for cancer cell metastasis? A review. Cancer Cell Int..

[136] Jiang J., Batra S., Zhang J. (2021). Asparagine: A metabolite to be targeted in cancers. Metabolites.

[137] Glunde K., Bhujwalla Z.M., Ronen S.M. (2011). Choline metabolism in malignant transformation. Nat. Rev. Cancer..

[138] Sen S., Kawahara B., Mahata S.K., Tsai R., Yoon A., Hwang L., Hu-Moore K., Villanueva C., Vajihuddin A., Parameshwar P. (2016). Cystathionine: A novel oncometabolite in human breast cancer. Arch. Biochem. Biophys..

[139] Ajouz H., Mukherji D., Shamseddine A. (2014). Secondary bile acids: An underrecognized cause of colon cancer. World J. Surg. Oncol..

[140] Paz E.A., LaFleur B., Gerner E.W. (2014). Polyamines are oncometabolites that regulate the LIN28/let-7 pathway in colorectal cancer cells. Mol. Carcinog..

[141] Rodriguez A.C., Blanchard Z., Maurer K.A., Gertz J. (2019). Estrogen signaling in endometrial cancer: A key oncogenic pathway with several open questions. Horm. Cancer.

[142] Yang M., Soga T., Pollard P.J., Adam J. (2012). The emerging role of fumarate as an oncometabolite. Front. Oncol..

[143] Agopian J., Da Costa Q., Nguyen Q.V., Scorrano G., Kousteridou P., Yuan M., Chelbi R., Goubard A., Castellano R., Maurizio J. (2021). GlcNAc is a mast-cell chromatin-remodeling oncometabolite that promotes systemic mastocytosis aggressiveness. Blood.

[144] Hochwald J.S., Zhang J. (2017). Glucose oncometabolism of esophageal cancer. Anticancer Agents Med. Chem..

[145] Choi Y.K., Park K.G. (2018). Targeting glutamine metabolism for cancer treatment. Biomol. Ther..

[146] Shim E.H., Livi C.B., Rakheja D., Tan J., Benson D., Parekh V., Kho E.Y., Ghosh A.P., Kirkman R., Velu S. (2014). L-2-Hydroxyglutarate: An epigenetic modifier and putative oncometabolite in renal cancer. Cancer Discov..

[147] Beyoğlu D., Idle J.R. (2021). Metabolic rewiring and the characterization of oncometabolites. Cancers.

[148] Hasan T., Arora R., Bansal A.K., Bhattacharya R., Sharma G.S., Singh L.R. (2019). Disturbed homocysteine metabolism is associated with cancer. Exp. Mol. Med..

[149] Gao P., Yang C., Nesvick C.L., Feldman M.J., Sizdahkhani S., Liu H., Chu H., Yang F., Tang L., Tian J. (2016). Hypotaurine evokes a malignant phenotype in glioma through aberrant hypoxic signaling. Oncotarget.

[150] Sivanand S., Vander Heiden M.G. (2020). Emerging roles for branched-chain amino acid metabolism in cancer. Cancer Cell..

[151] Venkateswaran N., Conacci-Sorrell M. (2020). Kynurenine: An oncometabolite in colon cancer. Cell Stress.

[152] De la Cruz-López K.G., Castro-Muñoz L.J., Reyes-Hernández D.O., García-Carrancá A., Manzo-Merino J. (2019). Lactate in the regulation of tumor microenvironment and therapeutic approaches. Front. Oncol..

[153] Wanders D., Hobson K., Ji X. (2020). Methionine restriction and cancer biology. Nutrients.

[154] Nokin M.J., Bellier J., Durieux F., Peulen O., Rademaker G., Gabriel M., Monseur C., Charloteaux B., Verbeke L., van Laere S. (2019). Methylglyoxal, a glycolysis metabolite, triggers metastasis through MEK/ERK/SMAD1 pathway activation in breast cancer. Breast Cancer Res..

[155] Forny P., Hochuli M., Rahman Y., Deheragoda M., Weber A., Baruteau J., Grunewald S. (2019). Liver neoplasms in methylmalonic aciduria: An emerging complication. J. Inherit. Metab Dis..

[156] Lala P.K., Chakraborty C. (2001). Role of nitric oxide in carcinogenesis and tumour progression. Lancet Oncol..

[157] Mierzejewska P., Kunc M., Zabielska-Kaczorowska M.A., Kutryb-Zajac B., Pelikant-Malecka I., Braczko A., Jablonska P., Romaszko P., Koszalka P., Szade J. (2021). An unusual nicotinamide derivative, 4-pyridone-3-carboxamide ribonucleoside (4PYR), is a novel endothelial toxin and oncometabolite. Exp. Mol. Med..

[158] Patel R., Raj A.K., Lokhande K.B., Almasri M.A., Alzahrani K.J., Almeslet A.S., Swamy K.V., Sarode G.S., Sarode S.C., Patil S. (2021). Detection of nail oncometabolite SAICAR in oral cancer patients and its molecular interactions with PKM2 enzyme. Int. J. Environ. Res. Public Health.

[159] Kanaan Y.M., Sampey B.P., Beyene D., Esnakula A.K., Naab T.J., Ricks-Santi L.J., Dasi S., Day A., Blackman K.W., Frederick W. (2014). Metabolic profile of triple-negative breast cancer in African-American women reveals potential biomarkers of aggressive disease. Cancer Genom. Proteom..

[160] Amelio I., Cutruzzolá F., Antonov A., Agostini M., Melino G. (2014). Serine and glycine metabolism in cancer. Trends Biochem. Sci..

[161] Nowicki S., Gottlieb E. (2015). Oncometabolites: Tailoring our genes. FEBS J..

[162] Yang F., Li J., Deng H., Wang Y., Lei C., Wang Q., Xiang J., Liang L., Xia J., Pan X. (2019). GSTZ1-1 Deficiency activates NRF2/IGF1R axis in HCC via accumulation of oncometabolite succinylacetone. EMBO J..

[163] Mi S., Gong L., Sui Z. (2020). Friend or foe? An unrecognized role of uric acid in cancer development and the potential anticancer effects of uric acid-lowering drugs. J. Cancer.

[164] Croteau E., Renaud J.M., Richard M.A., Ruddy T.D., Bénard F., deKemp R.A. (2016). PET metabolic biomarkers for cancer. Biomark. Cancer.

[165] Bamji-Stocke S., van Berkel V., Miller D.M., Frieboes H.B. (2018). A review of metabolism-associated biomarkers in lung cancer diagnosis and treatment. Metabolomics.

[166] Erben V., Bhardwaj M., Schrotz-King P., Brenner H. (2018). Metabolomics biomarkers for detection of colorectal neoplasms: A systematic review. Cancers.

[167] Dinges S.S., Hohm A., Vandergrift L.A., Nowak J., Habbel P., Kaltashov I.A., Cheng L.L. (2019). Cancer metabolomic markers in urine: Evidence, techniques and recommendations. Nat. Rev. Urol..

[168] Simińska E., Koba M. (2016). Amino acid profiling as a method of discovering biomarkers for early diagnosis of cancer. Amino Acids..

[169] Lee S.H., Mahendran R., Tham S.M., Thamboo T.P., Chionh B.J., Lim Y.X., Tsang W.C., Wu Q.H., Chia J.Y., Tay M.H.W. (2021). Tryptophan-kynurenine ratio as a biomarker of bladder cancer. BJU Int..

[170] Wang W., Tian S.L., Jin D., Liu B., Wang W., Chang H., Chen C., Yu Z., Wang Y.Z., Li Y.L. (2021). The role of bile acid subtypes in the diagnosis of cholangiocarcinoma. Asia Pac. J. Clin. Oncol..

[171] Hu X., Walker D.I., Liang Y., Smith M.R., Orr M.L., Juran B.D., Ma C., Uppal K., Koval M., Martin G.S. (2021). A scalable workflow to characterize the human exposome. Nat. Commun..

[172] Chung M.K., Kannan K., Louis G.M., Patel C.J. (2018). Toward capturing the exposome: Exposure biomarker variability and coexposure patterns in the shared environment. Environ. Sci. Technol..

[173] Sexton K., Adgate J.L., Fredrickson A.L., Ryan A.D., Needham L.L., Ashley D.L. (2006). Using biologic markers in blood to assess exposure to multiple environmental chemicals for inner-city children 3–6 years of age. Environ. Health Perspect..

[174] Kirman C.R., Aylward L.L., Blount B.C., Pyatt D.W., Hays S.M. (2012). Evaluation of NHANES biomonitoring data for volatile organic chemicals in blood: Application of chemical-specific screening criteria. J. Expo. Sci. Environ. Epidemiol..

[175] Dresen S., Ferreirós N., Gnann H., Zimmermann R., Weinmann W. (2010). Detection and identification of 700 drugs by multi-target screening with a 3200 Q TRAP LC-MS/MS system and library searching. Anal. Bioanal. Chem..

[176] Rappaport S.M., Barupal D.K., Wishart D., Vineis P., Scalbert A. (2014). The blood exposome and its role in discovering causes of disease. Environ. Health Perspect..

[177] Wang A., Gerona R.R., Schwartz J.M., Lin T., Sirota M., Morello-Frosch R., Woodruff T.J. (2018). A suspect screening method for characterizing multiple chemical exposures among a demographically diverse population of pregnant women in San Francisco. Environ. Health Perspect..

[178] Walker D.I., Juran B.D., Cheung A.C., Schlicht E.M., Liang Y., Niedzwiecki M., LaRusso N.F., Gores G.J., Jones D.P., Miller G.W. (2021). High-resolution exposomics and metabolomics reveals specific associations in cholestatic liver diseases. Hepatol. Commun..

[179] Kowalska G. (2021). The safety assessment of toxic metals in commonly used herbs, spices, tea, and coffee in Poland. Int. J. Environ. Res. Public Health..

[180] Li X., Tian T., Shang X., Zhang R., Xie H., Wang X., Wang H., Xie Q., Chen J., Kadokami K. (2020). Occurrence and health risks of organic micro-pollutants and metals in groundwater of Chinese rural areas. Environ. Health Perspect..

[181] Maruvada P., Lampe J.W., Wishart D.S., Barupal D., Chester D.N., Dodd D., Djoumbou-Feunang Y., Dorrestein P.C., Dragsted L.O., Draper J. (2020). Perspective: Dietary biomarkers of intake and exposure-exploration with omics approaches. Adv. Nutr..

[182] Dragsted L.O., Gao Q., Scalbert A., Vergères G., Kolehmainen M., Manach C., Brennan L., Afman L.A., Wishart D.S., Andres Lacueva C. (2018). Validation of biomarkers of food intake-critical assessment of candidate biomarkers. Genes Nutr..

[183] Loftfield E., Stepien M., Viallon V., Trijsburg L., Rothwell J.A., Robinot N., Biessy C., Bergdahl I.A., Bodén S., Schulze M.B. (2021). Novel biomarkers of habitual alcohol intake and associations with risk of pancreatic and liver cancers and liver disease mortality. J. Natl. Cancer Inst..

[184] Schmidt J.A., Fensom G.K., Rinaldi S., Scalbert A., Gunter M.J., Holmes M.V., Key T.J., Travis R.C. (2021). NMR metabolite profiles in male meat-eaters, fish-eaters, vegetarians and vegans, and comparison with MS metabolite profiles. Metabolites.

[185] Rothwell J.A., Murphy N., Bešević J., Kliemann N., Jenab M., Ferrari P., Achaintre D., Gicquiau A., Vozar B., Scalbert A. (2020). Metabolic signatures of healthy lifestyle patterns and colorectal cancer risk in a european cohort. Clin. Gastroenterol. Hepatol..

[186] Chen C.S., Kuo T.C., Kuo H.C., Tseng Y.J., Kuo C.H., Yuan T.H., Chan C.C. (2019). Metabolomics of children and adolescents exposed to industrial carcinogenic pollutants. Environ. Sci. Technol..

[187] Chen C.S., Kuo T.C., Kuo H.C., Tseng Y.J., Kuo C.H., Yuan T.H., Chan C.C. (2021). Lipidomics of children and adolescents exposed to multiple industrial pollutants. Environ. Res..

[188] Wang Z., Zheng Y., Zhao B., Zhang Y., Liu Z., Xu J., Chen Y., Yang Z., Wang F., Wang H. (2015). Human metabolic responses to chronic environmental polycyclic aromatic hydrocarbon exposure by a metabolomic approach. J. Proteome Res..

[189] Orešič M., McGlinchey A., Wheelock C.E., Hyötyläinen T. (2020). Metabolic signatures of the exposome-quantifying the impact of exposure to environmental chemicals on human health. Metabolites.

[190] Bessonneau V., Rudel R.A. (2019). Mapping the human exposome to uncover the causes of breast cancer. Int. J. Environ. Res. Public Health.

[191] Deng L., Chang D., Foshaug R.R., Eisner R., Tso V.K., Wishart D.S., Fedorak R.N. (2017). Development and validation of a high-throughput mass spectrometry based urine metabolomic test for the detection of colonic adenomatous polyps. Metabolites.

[192] Tsoli M., Daskalakis K., Kassi E., Kaltsas G., Tsolakis A.V. (2021). A critical appraisal of contemporary and novel biomarkers in pheochromocytomas and adrenocortical tumors. Biology.

[193] Weber D.D., Aminzadeh-Gohari S., Tulipan J., Catalano L., Feichtinger R.G., Kofler B. (2020). Ketogenic diet in the treatment of cancer—Where do we stand?. Mol. Metab..

[194] Maddocks O.D., Berkers C.R., Mason S.M., Zheng L., Blyth K., Gottlieb E., Vousden K.H. (2013). Serine starvation induces stress and p53-dependent metabolic remodelling in cancer cells. Nature.

[195] Brandhorst S., Longo V.D. (2016). Fasting and caloric restriction in cancer prevention and treatment. Recent results. Cancer Res..

[196] Clifton K.K., Ma C.X., Fontana L., Peterson L.L. (2021). Intermittent fasting in the prevention and treatment of cancer. CA Cancer J. Clin..

[197] Butler M., van der Meer L.T., van Leeuwen F.N. (2021). Amino acid depletion therapies: Starving cancer cells to death. Trends Endocrinol. Metab..

[198] Ribeiro M.D., Silva A.S., Bailey K.M., Kumar N.B., Sellers T.A., Gatenby R.A., Ibrahim-Hashim A., Gillies R.J. (2012). Buffer therapy for cancer. J. Nutr. Food Sci..

[199] Ibrahim-Hashim A., Abrahams D., Enriquez-Navas P.M., Luddy K., Gatenby R.A., Gillies R.J. (2017). Tris-base buffer: A promising new inhibitor for cancer progression and metastasis. Cancer Med..

[200] Yang M., Zhong X., Yuan Y. (2020). Does baking soda function as a magic bullet for patients with cancer? A mini review. Integr. Cancer Ther..

[201] Gonzalez C.A., Riboli E. (2010). Diet and cancer prevention: Contributions from the European Prospective Investigation into Cancer and Nutrition (EPIC) study. Eur. J. Cancer.

[202] Geijsen A.J.M.R., Brezina S., Keski-Rahkonen P., Baierl A., Bachleitner-Hofmann T., Bergmann M.M., Boehm J., Brenner H., Chang-Claude J., van Duijnhoven F.J.B. (2019). Plasma metabolites associated with colorectal cancer: A discovery-replication strategy. Int. J. Cancer.

[203] Weir T.L., Manter D.K., Sheflin A.M., Barnett B.A., Heuberger A.L., Ryan E.P. (2013). Stool microbiome and metabolome differences between colorectal cancer patients and healthy adults. PLoS ONE.

[204] Louis P., Hold G.L., Flint H.J. (2014). The gut microbiota, bacterial metabolites and colorectal cancer. Nat. Rev. Microbiol..

[205] Pham C.H., Lee J.E., Yu J., Lee S.H., Yu K.R., Hong J., Cho N., Kim S., Kang D., Lee S. (2021). Anticancer effects of propionic acid inducing cell death in cervical cancer cells. Molecules.

[206] Sreedhar A., Zhao Y. (2018). Dysregulated metabolic enzymes and metabolic reprogramming in cancer cells. Biomed. Rep..

[207] Liu J., Zhang C., Hu W., Feng Z. (2019). Tumor suppressor p53 and metabolism. J. Mol. Cell. Biol..

[208] Huang R., Liu X., Li H., Zhou Y., Zhou P.K. (2020). Integrated analysis of transcriptomic and metabolomic profiling reveal the p53 associated pathways underlying the response to ionizing radiation in HBE cells. Cell. Biosci..

[209] Zhu Y., Piao C., Zhang Z., Jiang Y., Kong C. (2021). The potential role of c-MYC and polyamine metabolism in multiple drug resistance in bladder cancer investigated by metabonomics. Genomics.

[210] Dogra R., Bhatia R., Shankar R., Bansal P., Rawal R.K. (2018). Enasidenib: First mutant IDH2 inhibitor for the treatment of refractory and relapsed acute myeloid leukemia. Anticancer Agents Med. Chem..

[211] Roboz G.J., DiNardo C.D., Stein E.M., de Botton S., Mims A.S., Prince G.T., Altman J.K., Arellano M.L., Donnellan W., Erba H.P. (2020). Ivosidenib induces deep durable remissions in patients with newly diagnosed IDH1-mutant acute myeloid leukemia. Blood.

[212] Oh S., Cho Y., Chang M., Park S., Kwon H. (2021). Metformin decreases 2-HG production through the MYC-PHGDH pathway in suppressing breast cancer cell proliferation. Metabolites.

